# An ancient alpharetrovirus lineage in bats: Evolutionary insights and possible roles in reproduction

**DOI:** 10.1371/journal.pgen.1012277

**Published:** 2026-08-25

**Authors:** Emma F. Harding, Zhaobo Zhang, Charles Reuben De Souza, Jose Gabriel Nino Barreat, Aris Katzourakis

**Affiliations:** Department of Biology, University of Oxford, United Kingdom; Fred Hutchinson Cancer Research Center, UNITED STATES OF AMERICA

## Abstract

Alpharetroviruses are an important group of pathogens known to cause leukemias and tumors, and were historically considered to be restricted to avian hosts. The identification of alpharetrovirus-like envelopes in bat genomes has hinted at a potentially wider host range, although their relations to modern alpharetroviruses and distribution remains unclear. Through a paleovirological screening of 818 vertebrate genomes we identified CHIRalphaEnv, a lineage that belongs firmly within alpharetroviruses, and emerged from a cross-class transmission from saurian hosts. We determine that CHIRalphaEnv envelope genes have been co-opted across bats on eight separate occasions between 43.8 and 18.9 million years ago and are preserved in most bat genomes screened. CHIRalphaEnv elements encode full-length envelope proteins and have been maintained under purifying selection, demonstrating multiple instances of exaptation by their bat hosts and a likely ubiquitous function. We observe high expression levels of CHIRalphaEnv envelopes in endometrium tissue from *Carollia perspicillata,* suggesting an involvement in reproductive function. We find CHIRalphaEnv sequence relatives in multiple mammalian clades (Afrotherians, rodents and bats), expanding the host range and extending origins of alpharetroviruses beyond 43 million years. We also propose the first mammalian co-opted Endogenous retrovirus (ERV) derived from an *Alpharetrovirus* envelope and explore the convergent functional recruitment of CHIRalphaEnv in hemochorial placentation in bats, elephant shrews and spiny mice. These findings highlight alpharetroviruses as a previously underappreciated source of functional exaptation in mammals.

## Author summary

Retroviruses are important pathogens, providing constant selective pressure and sources of genetic variability throughout vertebrate evolution. Significant evolutionary leaps have been instigated by retroviral genes, such as syncytin that has been domesticated by the host and repurposed for roles in reproduction. This study identifies eight related domestication events of a retroviral envelope gene that occurred between approximately 19–44 million years ago in bats. Its remarkable conservation over time and ubiquitous presence in all bat lineages implies that it was repurposed for a vital host function. We find evidence of its activity in endometrium cells during pregnancy, suggesting that it plays a contemporary role in bat reproduction and may be involved in the evolution of placentation. Intriguingly, the lineage of viruses that gave rise to this domesticated envelope (alpharetroviruses) is a bird pathogen today and has not been observed to infect bats. Through tracing its evolutionary history, we show that there has been at least one historical jump of these alpharetroviruses from reptiles to mammals. In summary, we explore a new lineage of retroviruses that have successfully jumped between hosts and contributed to bat evolution through time. This has broad implications for our understanding of retroviral zoonosis, mammalian evolution and virus-host interactions.

## Introduction

*Retroviridae* is a diverse family of RNA viruses that have coevolved with vertebrates for hundreds of millions of years [1]. Throughout this time, retroviruses have influenced the evolution of their vertebrate hosts by horizontally transferring genes, shaping immune responses and inducing genome rearrangements [2–6]. The key factor that has enabled their impacts on vertebrates is their obligate integration into host chromosomal DNA. When this integration occurs in germline cells, retroviral DNA is passed onto progeny and can persist in a population until it either becomes fixed or is lost. Hundreds of millions of years of retroviral infections have led to the accumulation of endogenous retroviruses (ERVs) within animal genomes that can be used as a molecular “fossil record” to trace retroviral evolution.

Classification of an ERV is based primarily on the polymerase gene, as retroviruses frequently recombine to acquire new envelopes which can complicate classification. Modern retroviruses are widely distributed across vertebrates and are classified into fourteen genera which are encompassed in three major clades: Clade I comprises *Gammaretrovirus* and *Epsilonretrovirus,* Clade II comprises *Alpharetrovirus, Betaretrovirus, Deltaretrovirus* and *Lentivirus,* and Clade III comprises *Spumaretrovirinae* [7]*.* ERVs of all clades have been described, however their prevalence varies greatly. For example, *Gammaretrovirus* ERVs are ubiquitously present in vertebrate genomes whilst *Lentivirus* ERVs are much rarer and have only been described in select species of mammals [8–14]. *Alpharetrovirus* ERVs are particularly scarce and are the only genus not yet identified in mammalian genomes, existing exclusively in avian genomes [14–16].

Although *Alpharetrovirus* polymerase sequences have only been recovered from avian hosts, alpharetrovirus-like envelope elements (AREs) have been reported in amphibian, reptile, monotreme and bat genomes [17–19]. Retroviral envelopes often have distinct evolutionary histories to polymerases, recombining indiscriminately to confer new host ranges and physiochemical properties to a retroviral lineage.

The presence of AREs in bat genomes is of particular interest, given the absence of characterized *Alpharetrovirus* ERVs in mammals, and raises questions about the evolutionary history, transmission and persistence of alpharetroviruses in bats [19,20]. AREs have been detected in eleven bat families spanning much of the known bat diversity, indicating they were prevalent viral pathogens within the order Chiroptera [20]. These findings pose many new questions: namely are these bat AREs true members of the *Alpharetrovirus* genus that once circulated more widely or are these AREs simply their closest endogenous relatives.

Retroviral envelopes are a crucial interface between the virus and the host cell and play an important role in the retroviral replication cycle, mediating cell entry, cell-cell fusion and immunosuppression [21,22]. The mechanisms of envelope interactions can be incidentally useful in other processes, performing functions that confer a benefit for the host and has led to endogenous envelopes being frequently co-opted.

The most well-studied example of retroviral envelope co-option are syncytin genes, which play essential roles in placental morphogenesis and reproduction, contributing to formation of the syncytiotrophoblast – the placental barrier between the foetus and mother [23,24]. Remarkably, syncytin co-option has occurred on at least nine separate occasions throughout mammals, with distinct host lineages carrying entirely independent retroviral genes that perform this function, including instances of sequential replacement of the ancestral syncytin with more recently co-opted envelopes through evolution [23,25,26]. A syncytin has also been identified in the viviparous *Mabuya* lizard, demonstrating that this phenomenon is not restricted to mammals and can occur convergently in vertebrates [17]. Some syncytins affect other physiological processes aside from reproduction: mice syncytins contribute to cell fusion during muscle formation and repair and subsequently impact muscle sexual dimorphism [27]. The convergent capture of retroviral envelopes and their involvement in diverse physiological functions are a testament to their evolutionary success.

Aside from their role in key physiological processes, retroviral envelopes have also been co-opted for antiviral defence against exogenous viral infection. A common mechanism for co-opted envelopes to restrict viral infection is through receptor interference, whereby expression of endogenous envelope saturates cellular receptors and subsequently blocks the binding of infecting virions [2]. This antiviral strategy has been observed in multiple vertebrates and is effective against diverse retroviral genera: the *Fv-4* gene in mice restricts the gammaretrovirus Murine leukemia virus [28], *ev3*, *ev6* and *ev9* genes in chickens restrict alpharetrovirus infection [29], and endogenous Jaagsiekte sheep retrovirus (enJSRV) restricts its exogenous betaretrovirus counterpart [30].

In this study we describe and characterise a lineage of alpharetrovirus-like envelope ERVs in Chiroptera*.* Using genomic analysis and phylogenetics, we establish whether this lineage belongs to the *Alpharetrovirus* genus and explore whether these elements are maintained within bat genomes through purifying selection indicating likely host function. We also reconstruct the evolutionary history of this viral lineage and determine if they were introduced into bats through cross-species transmission. By combining these observations, we explore the evolutionary history and investigate the possible functions of alpharetrovirus envelopes in bats.

## Results

### Conserved alpharetrovirus envelope elements within bats

**Fig 1:**
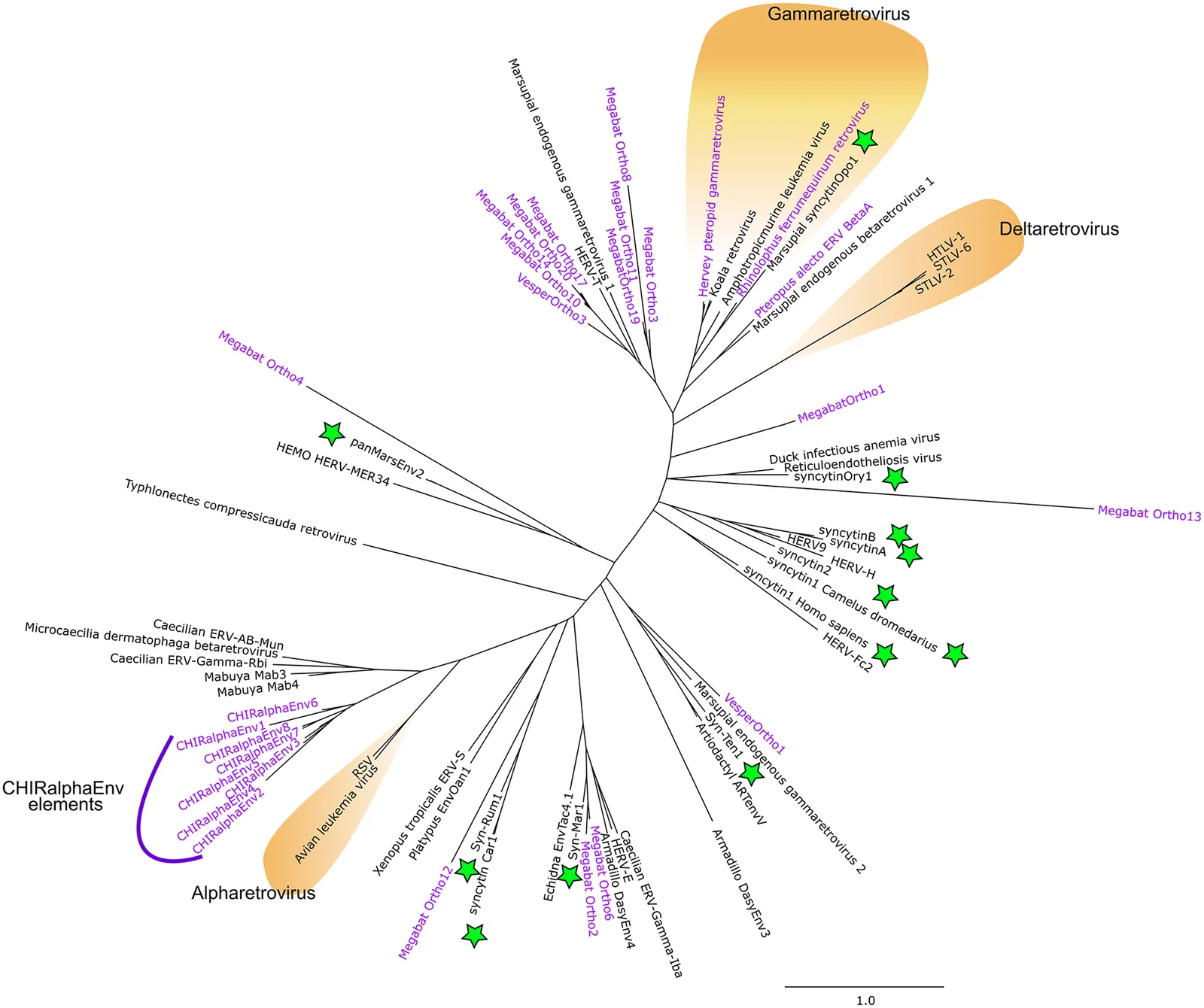
Phylogenetic relationship of CHIRalphaEnv elements to other retroviral envelopes and ERV elements. *Amino acid sequences of circulating retroviruses, envelope-derived endogenous elements and syncytins were compiled from NCBI databases and literature.* In silico *translated CHIRalphaEnv elements were aligned with these reference sequences using MAFFT, trimmed with TRIMal and used to construct a phylogenetic tree with IQTREE2 with 1000 ultrafast bootstraps using the WAG + F + R5 model. Boostrap supports >70 are shown. Viruses with known bat tropism are coloured purple. Green stars indicate co-opted syncytins. Endogenous bat sequences were obtained from the following studies: Megabat and vespertilionid orthogroups and* Rhinolophus *alpha-like [*19*] and megabat EVEs [*32*].*

**Fig 2:**
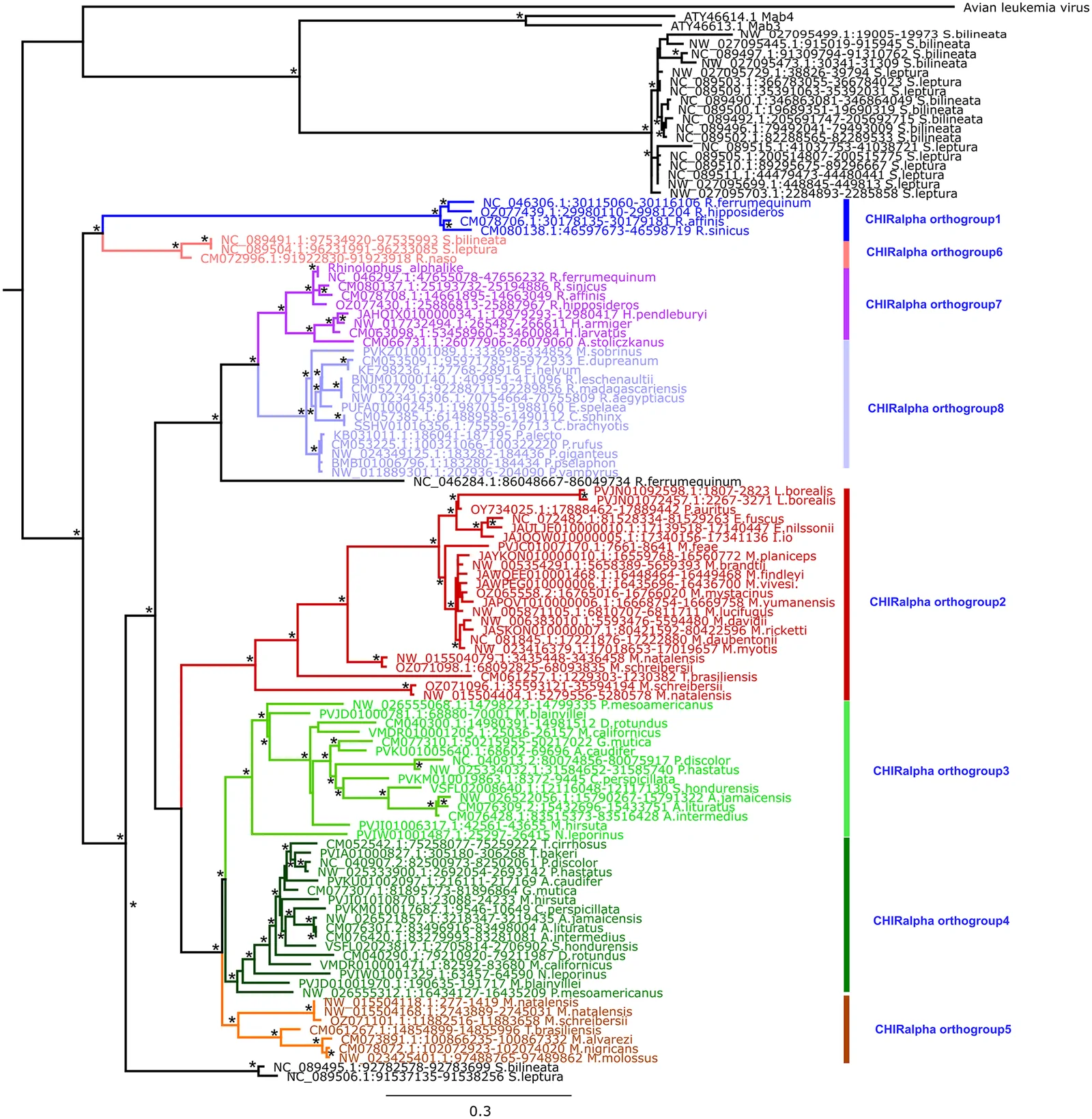
Phylogenetic relationships between orthologous clades of CHIRalphaEnv elements within chiropteran genomes. In silico translated CHIRalphaEnv elements were aligned using MAFFT, trimmed with TRIMal and used to construct a phylogenetic tree with IQTREE2 with 1000 ultrafast bootstraps. Boostrap supports >70 are indicated with *.

To identify *Alpharetrovirus-*like envelopes (AREs) in bats, we screened 80 available chiropteran genomes using the BLAST-based workflow HI-FEVER [31] and validated hits with a custom Hidden Markov Model (HMM), available in the associated OSF repository. AREs were detected in 79/80 genomes comprising 2,739 total sequences of which 141 were intact and retained >300 aa ORFs, representing full or near full-length envelope genes (Tables A & B in S1 Appendix). Reciprocal BLASTx searches against the Reference Viral Database (RVDB) revealed that these elements had closest identity (29.9-40.0% over the 385 aa CDS) to the envelope proteins Mab3 and Mab4 in *Mabuya* lizards [17]. Phylogenetically they form a sister clade to endogenous elements from lizards and caecilians (97% bootstrap support), which themselves are a sister clade to modern *Alpharetrovirus* sequences in birds (100% bootstrap support) (Fig 1). The *Rhinolophus* alpha-like sequence previously reported [19] clustered with these bat AREs, indicating they are from the same retroviral lineage (Fig 2). The 141 intact elements split into two clades: one comprised exclusively of *Saccopteryx leptura* and *Saccopteryx bilineata* sequences (n = 39) that clusters with endogenous sequences from lizards and caecilians. The other clade comprises the remaining 102 sequences spanning all chiropteran species surveyed (Table C in S1 Appendix). We designated this clade CHIRalphaEnv (CHIRopteran alpharetrovirus envelope) and focussed on it for further analysis.

To ascertain if these intact CHIRalphaEnv were inherited from a single ancient integration in a common ancestor, or were remnants of multiple distinct integration events, we examined their chromosomal locations for orthology. Flanking regions (30 kb) of each CHIRalphaEnv element were compared to determine orthology. We identified eight main orthogroups (CHIRalphaEnv 1–8) encompassing 91 of the 102 intact CHIRalphaEnv elements >300 aa (Fig 2, Table D in S1 Appendix). The genomic location of each orthogroup was confirmed by identifying annotated synteny of each CHIRalphaEnv element.

Yinpterochiroptera CHIRalphaEnv elements grouped into three orthogroups: CHIRalphaEnv1 in the *Rhinolophus* genus, CHIRalphaEnv7 in the Pteropodidae family and CHIRalphaEnv8 in Rhinolophidae and Hipposeridae bats. CHIRalphaEnv1 elements (n = 4) were integrated between the *IL15* and *RNF150* genes. CHIRalphaEnv7 (n = 14) were integrated between the *PRXL2C* and *CDC14B* genes, and CHIRalphaEnv8 (n = 9) were integrated downstream of the *ABRA* gene.

Yangochiroptera CHIRalphaEnv elements grouped into five orthogroups. CHIRalphaEnv2 elements (n = 22) were present in bats from the Vespertilionidae family and located within an intron of the *NCOA7* gene. CHIRalphaEnv3 elements (n = 15) and CHIRalphaEnv5 elements (n = 17) were only detected in Phyllostomidae bats and located between the *LONRF1* and *TRMT9B*, and *GF0D1* and *SIRT5* genes respectively. CHIRalphaEnv4 elements (n = 7) were present in the Molossidae family and located within an intron of the *CALD1* gene. CHIRalphaEnv6 elements (n = 3) were present in Emballonuridae bats between the *ZNF169* and *PTPDC1* genes.

Interestingly, 25 bat species contained intact CHIRalphaEnv elements from multiple orthogroups (Fig 2 and Table A in S1 Appendix), indicating multiple co-option events within these genomes.

To identify other retroviral features surrounding CHIRalphaEnv elements we performed BLAST searches of the 20 kb surrounding each element. We identified remnants of retroviral *gag* and *pol* regions upstream of the *env* ORF, and in some cases long terminal repeats flanking the elements (Tables A & E in S1 Appendix). In all cases the *gag* and *pol* genes had degraded leaving only short (<75 aa) ORFs. A BLAST search revealed the highest match of the translated *pol* fragments of most (n = 7/8) orthogroups was ARTgagpol (33–60% aa identity), an env-less gammaretrovirus ERV identified in camels [33] (Table E in S1 Appendix). In contrast, CHIRalphaEnv1 *pol* fragments had reciprocal BLASTx identity (46.4% aa) to ERV-L elements and phylogenetically clustered with Clade III ERVs (S1 Fig).

### ERV dating and rate of LTR mutation in pteropodid genomes

We estimated the minimum integration time of each orthogroup based on their host divergence dates, obtained from timetree.org, and LTR divergence dating where possible. CHIRalphaEnv1, 2, 3, 4, 5 and 8 were the oldest, all integrating between 36.46 and 43.80 million years ago (Tables D & F in S1 Appendix and S2 Fig). CHIRalphaEnv6 and 7 are estimated to be younger, integrating between 18.87 and 28.42 million years ago (Tables D & F in S1 Appendix and S2 Fig). The estimated integration times between the youngest (18.87 my) and oldest (43.80my) elements indicate that the progenitor retroviral lineage circulated in bats for at least 24.93 million years.

Many CHIRalphaEnv proviruses were integrated in regions of the genome that are enriched with other transposable elements including the non-LTR retrotransposons long and short interspersed nuclear elements (LINEs/SINEs) and Merlin DNA transposons. CHIRalphaEnv6 had 148 direct repeats with identity to LINEs, SINEs and Merlin DNA transposons within the 30kb upstream and downstream flanks of the *env* ORF. CHIRalphaEnv2 had 28 LTR candidates flanking CHIRalphaEnv2 with identity to the Vespertilionidae*-*exclusive VES SINE elements [34]. In contrast, the only detectable LTRs flanking CHIRalphaEnv3 were 690 bp and surrounded a 23,691 bp “provirus”, a region much larger than a predicted provirus. This heterogeneity in LTR origin, length and proximity to CHIRalphaEnv elements complicated LTR divergence dating of each orthogroup and made it unfeasible to estimate a time for many using this method.

This heterogeneity was not only observed between orthogroups but also intra-orthogroup and was most obvious for CHIRalphaEnv7 in Pteropodidae bats where both their LTR lengths and identities varied greatly. All CHIRalphaEnv7 LTRs shared 81–85% similarity to RhiSin-5.3463, an annotated ERV LTR from *Rhinolophus sinicus* [35]*,* however their lengths varied: *Cynopterus* and *Rousettus* bats had the shortest LTRs (161–174 nt) whilst *Eidolon* bats had the longest (535–536 nt) (Table F in S1 Appendix). CHIRalphaEnv7 LTR identities ranged from 86.29% in *Cynopterus brachyotis* to 95.27% in *Macroglossus sobrinus* despite their identical integration times (Table C in S1 Appendix). The incongruency between LTR identities within orthologous ERVs means that their identity cannot be explained by a constant rate of neutral evolution over time, and their differing lengths suggests other factors like gene conversion or LTR homogenization may have also affected LTR evolution [36].

We used the LTR divergences and fixed common integration time of all CHIRalphaEnv7 elements to estimate the rate of neutral LTR mutation in Pteropodidae. With the given minimum integration time of 29.7 my between *Cynopterus* and *Pteropus* [37], we estimated an average substitution rate of 4.76855x10^-10^ substitutions/site/year. Amongst pteropids, *Cynopterus* and *Eidolon* bats had the fastest substitution rates, whilst *Pteropus* and *Rousettus* had the slowest. This estimated pteropodid rate of neutral LTR mutation is slower than the previously reported megabat rates of 1.9-2.6x10^-9^ substitutions/site/year [38,39]. When applying the megabat neutral rates to CHIRalphaEnv7 we estimated integration dates as recent as 3.31 million years ago which is inconsistent with the divergence time of the hosts (~29.7 million years) [37] (Table F in S1 Appendix). This suggests that firstly, CHIRalphaEnv LTRs evolve at different rates in different pteropodids, and secondly, that the megabat neutral rates of substitution does not accurately model the rate of LTR mutation in Pteropodidae ERVs.

### Motifs and function

**Fig 3:**
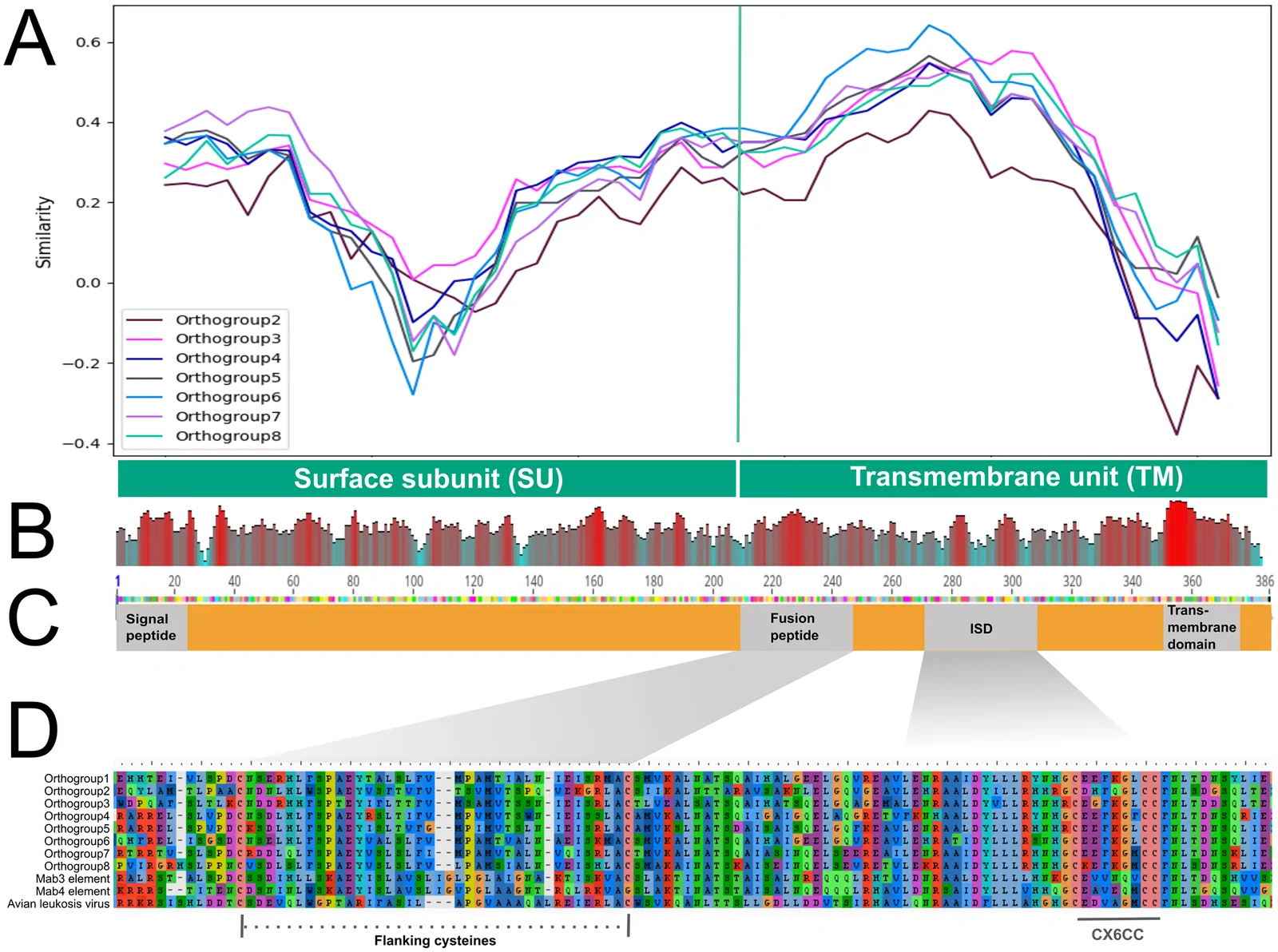
Characteristics of CHIRalphaEnv elements using the *Pteropus alecto* sequence as a reference. *A: Nucleotide similarity plots along the CHIRalphaEnv envelope ORF. Sequences from the eight CHIRalphaEnv orthogroups were aligned using MAFFT and analysed in SIMPLOT++ to generate the similarity plot. B: Hydrophobicity plot of amino acids along the CHIRalphaEnv envelope ORF. C: Organisation of the CHIRalphaEnv envelope ORF. The orange bar denotes the ORF, with grey rectangles denoting hallmark domains. D: Alignment of CHIRalphaEnv orthogroups showing the fusion peptide and immunosuppressive domain (ISD).* In silico *translated CHIRalphaEnv sequences were aligned with related proteins using MAFFT and visualised in AliView.*

We searched for characterised peptide motifs involved in canonical envelope functions. All CHIRalphaEnv orthogroups retained retroviral envelope hallmarks in the transmembrane (TM) domain including an immunosuppressive domain (ISD) and transmembrane region (Fig 3, Panels B & C). The representative of each orthogroup, with the exception of CHIRalphaEnv2 in Vespertilionidae, retained a 16–32 aa signal peptide at the N terminus of the CDS (Fig 3, Panel D). The canonical furin cleavage site RXXR was not consistently retained throughout any orthogroup except orthogroup 6 (Fig 3).

On the peptide level, CHIRalphaEnv elements shared unique characteristics of avian *Alpharetrovirus* sequences, including flanking cysteines around the fusion peptide and the lack of a CXXC motif utilized for covalent linkage of the envelope in the subunit (SU) domain (Fig 3, Panel D).

To investigate the possibility of convergent evolution for a common function, we compared nucleotide identity across the base positions of each orthogroup (Fig 3, Panel A). CHIRalphaEnv1 was selected as the reference outgroup due to its evolutionary distance from the other orthogroups (Fig 2). In general, orthogroups shared nucleotide identity over the TM domain more than the SU domain, with higher identity (40–60%) shared across the fusion peptide and ISD (Fig 3). This suggests that any conserved functions are likely to arise from those more-constrained TM domains rather than the more variable SU region.

### Selection analysis

We compared the rates of nonsynonomous to synonymous (d_N_/d_S_ or ω) mutations within the CHIRalphaEnv ORF. Values of this ratio where ω < 1 or ω > 1 indicate negative (purifying) selection and positive (diversifying) selection respectively, while ω = 1 cannot reject neutrality.

We firstly tested if the entire CHIRalphaEnv tree, comprising all eight CHIRalphaEnv orthogroups, was under purifying selection which would be suggestive of co-option and a conserved host function. Under the M0 one-ratio model, ω = 0.333 which indicates that the overall pattern of selection across the CHIRalphaEnv tree is purifying. Through comparison of one-ratio (M0, null hypothesis) and nearly neutral (M1a) site models, we observe that the M1 model fit the data better (M0 vs M1a 2Δl = 1,241.8), indicating that the selective pressure varies across sites of CHIRalphaEnv and cannot be accurately estimated with a single ω ratio across the ORF.

To test whether there was positive selection on any of the sites within the CHIRalphaEnv ORF, we compared models of nearly neutral selection (M1a/M7) and positive selection (M2a/M8) to observe which model best fit the observed sequences. Both the M2a and M8 models fit the data better than the M1a and M7 respectively, indicating the presence of site-specific positive selection. Four positions show significant signal of positive selection (>95% probability that ω > 1), two of which are around the SU/TM junction (sites 206/208) and two of which are in the cytoplasmic tail beyond the transmembrane domain (sites 378/389) (Table G in S1 Appendix). At these four sites we observe no preference for hydrophobic/hydrophilic or polar/non-polar residues, indicating a relaxation of structural and/or functional restraints on these residues.

We ran the tests of nearly neutral vs. positive site tests on each orthogroup to determine if they were influenced by the same selection patterns. Six of the eight orthogroups showed no significant selection when compared to the overall CHIRalphaEnv selection patterns (Table G in S1 Appendix). CHIRalphaEnv4 had one site (site 88, T - > F) which was under significant (>95% probability that ω > 1) positive selection when compared to the rest of the CHIRalphaEnv sequences. In contrast, the selection on CHIRalphaEnv1 sites differed greatly from the selection on other CHIRalphaEnv sequences. An estimated 14% of sites in CHIRalphaEnv1 were under diversifying selection whilst the same sites were under purifying selection in the other orthogroups. Seven of these sites were significant (>95% probability that ω > 1), one of which was more significant (>99% probability that ω > 1) (Table G in S1 Appendix). This suggests that the *Rhinolophus* orthogroup is under different selection pressures to the other CHIRalphaEnv orthogroups and may have been co-opted or since evolved for a different host function.

### Transcriptomic analysis

To search for transcription and therefore possible host function, we screened 1,982 bat RNA-Seq experiments from a range of bat species (n = 110) and tissues (35 categories) (Table H in S1 Appendix).

**Fig 4:**
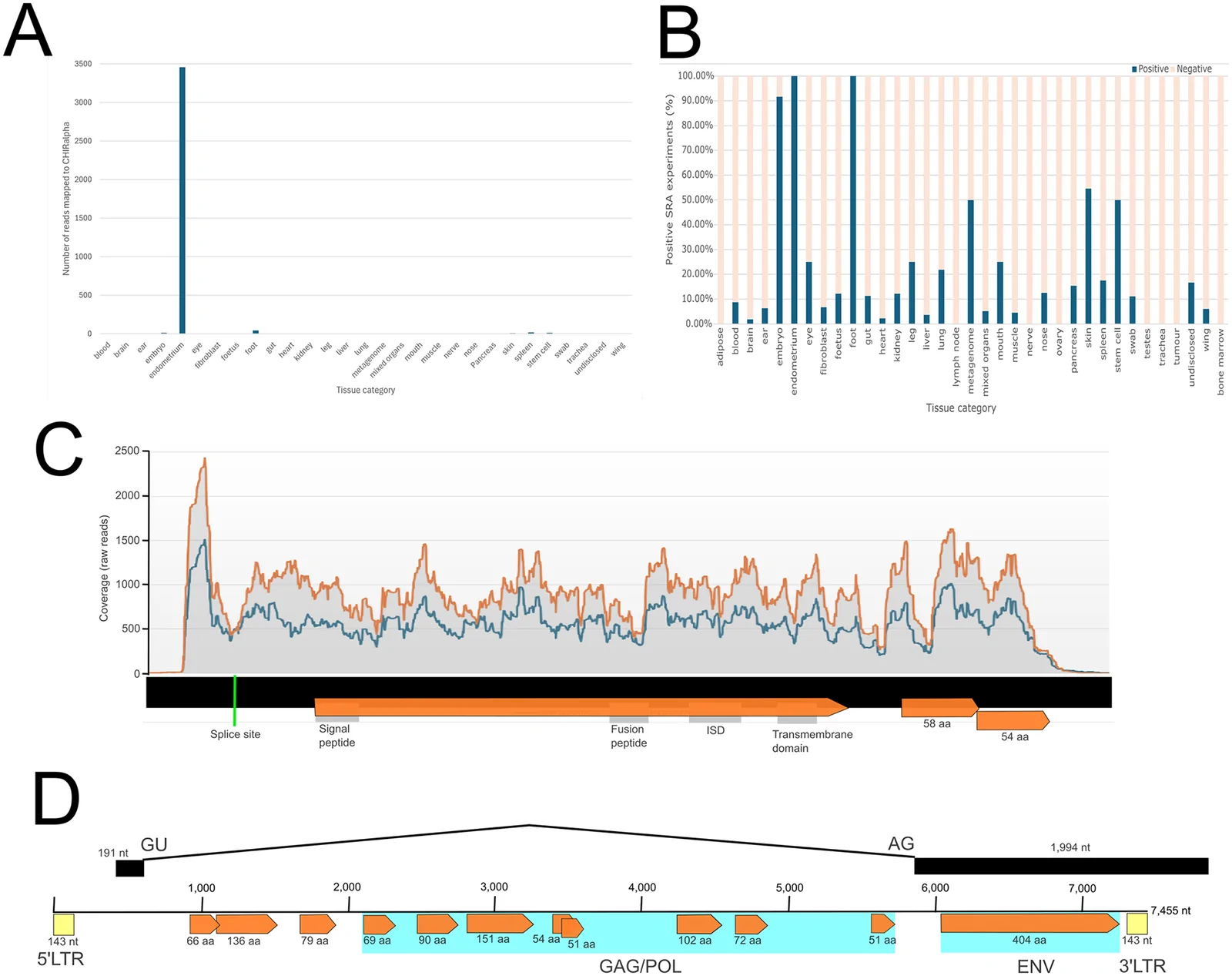
RNA-expression of the CHIRalphaEnv element in bats. *A: Normalised reads mapping to CHIRalphaEnv sequences in 1,982 bat SRA experiments, grouped by tissue type. B: Percentage of SRA experiments positive for expression of CHIRalphaEnv sequences from 1,982 SRA experiments. C:* Carollia perspicillata *CHIRalphaEnv transcript showing the splice site (green), ORFs (orange) and notable protein domains (grey) and mapped raw read coverage from two endometrium samples (SRR12330753 and SRR12330754 in orange and blue respectively). D: Map of the CHIRalphaEnv proviral element in* Carollia perspicillata *showing the production of spliced CHIRalphaEnv transcript. Proviral features are shaded as follows: long terminal repeats (yellow), ORFs (orange) and protein-coding regions (blue). The black bar above represents the spliced transcripts, with predicted splice sites shown.*

Of these 1,932 RNA datasets, 186 transcriptomes (9.38%) were positive for CHIRalphaEnv expression, defined as containing >5 raw reads with 100% identity to a bat CHIRalphaEnv. Most tissues exhibited low or no expression with the exception of embryonic cells (n = 12), endometrium (n = 2), foot (n = 2), skin (n = 11), stem cells (n = 6) and metagenome samples (n = 2) which had > 50% of samples positive (Fig 4 Panel B). Of these, both endometrium (n = 2) and foot tissue (n = 2) had a 100% positivity rate, suggesting it is highly expressed in these tissues and conditions: the two foot samples were from a *Myotis myotis* bat infected with the fungus (*Pseudogymnoascus destructans)* that causes white-nose syndrome and the two endometrium samples were from a pregnant *Carollia perspicillata* bat.

To quantify the level of RNA expression, the number of CHIRalphaEnv-mapping reads were normalized against the *EEF1A1* housekeeping gene reads for each positive RNA-Seq experiment. The embryonic cell experiments contained significantly higher read levels compared to other tissues, with an average of 0.001% of the total reads mapping to CHIRalphaEnv (Fig 4 Panel A & Table H in S1 Appendix). Expression levels were the highest in the two endometrium samples from pregnant *Carollia* bats with 0.007% and 0.009% respectively of total reads mapping to CHIRalphaEnv (Table H in S1 Appendix).

The two *Carollia* endometrium experiments were assembled into transcripts and aligned to the *Carollia* CHIRalphaEnv provirus locus. Of the two intact CHIRalphaEnv elements in the *Carollia perpicillata* genome, the endometrium transcripts originated from the orthogroup 5 element (PVKM010017682.1:9546–10649). No evidence of transcription from the orthogroup 3 element was observed. The CHIRalphaEnv transcript was a splice product of the genomic CHIRalphaEnv locus, comprising 191 nt of the provirus upstream of the *gag* and 1,994 nt of the end of the provirus encompassing the *env* ORF and downstream (Fig 4 Panels C & D). This is consistent with gamma- and alpharetrovirus spliced transcripts that produce *env* mRNA through removal of the *gag*/*pol* region (Fig 4 Panels C & D). Interestingly, the spliced transcript also contained two short (58 and 54 aa) ORFs downstream of the CHIRalphaEnv ORF which had no significant (E-value <0) BLAST similarity to proteins in the NCBI nr database.

To explore the possible fusogenic function of CHIRalphaEnv elements, we expressed CHIRalphaEnv orthogroups 2, 3, 4, 5 and 7 in human, bat and rodent cell lines under both normal conditions and after acid treatment (pH4). No synctyia were observed under these conditions, concordant with a loss of fusogenic activity (S3-S7 Figs).

### Evidence of cross-class transmission between reptiles and mammals

As no modern bat alpharetroviruses have been isolated, we aimed to trace the evolutionary history of the CHIRalphaEnv lineage and determine if they are “native” mammalian viruses that co-evolved with bats or the result of a cross-species transmission event. To determine the host range of CHIRalphaEnv elements, we performed a DIAMOND BLASTx search using the CHIRalphaEnv7 representative sequence to query available NCBI RefSeq vertebrate genomes (n = 738) (Table I in S1 Appendix). We identified 3,396 related elements in a variety of saurian (squamates, crocodilians, turtles, birds), amphibian (anurans, caecilians) and mammalian (afrotherians, rodents, lagomorphs) genomes (Table J in S1 Appendix).

**Fig 5:**
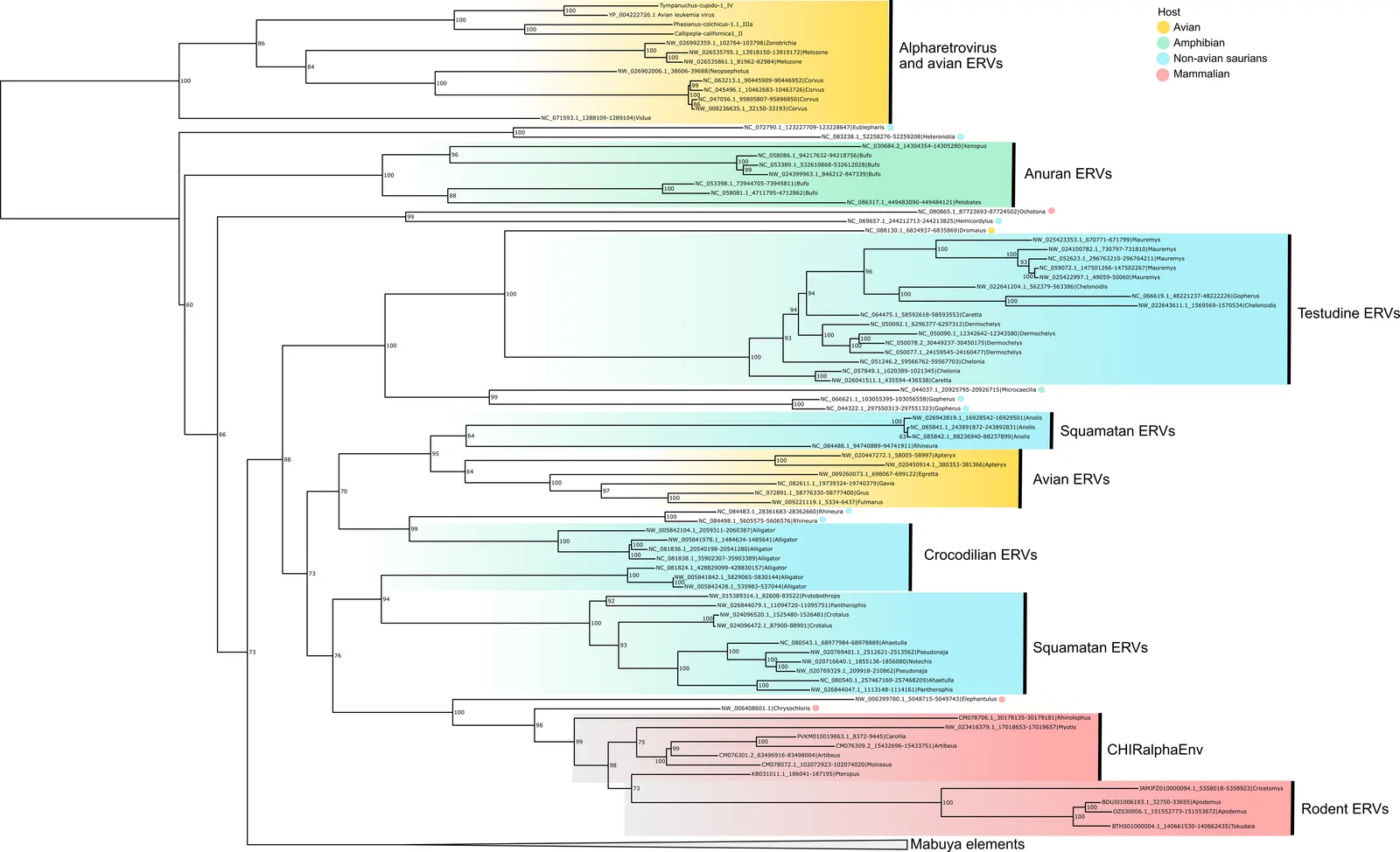
Phylogeny of CHIRalphaEnv and related intact alpharetrovirus-like envelopes in vertebrates. A DIAMOND-based search was used to identify CHIRalphaEnv-related sequences in all vertebrates. Intact sequences were in silico translated and aligned using MAFFT, trimmed with TRIMal and used to construct a phylogenetic tree with IQTREE2 with 1000 ultrafast bootstraps. Boostrap supports >70 are shown. Clades and nodes are shaded based on host classification: avian (yellow), amphibian (green), non-avian saurian (blue) and mammalian (red).

The closest CHIRalphaEnv relative is an intact ARE in a cape-golden mole (*Chrysochloris asiatica*), sharing 58.5% aa identity to CHIRalphaEnv7 over the 352 aa ORF. Other intact ORFs sharing >40% identity to CHIRalphaEnv sequences were present in the mammals: Okinawa spiny rat (*Tokudaia muenninki*, 45.7% identity), Cape elephant shrew (*Elephantulus edwardii*, 45.1% identity), large Japanese field mouse (*Apodemus speciosus*, 44.0% identity), striped field mouse (*Apodemus agrarius*, 43.0% identity) and Southern giant pouched rat (*Cricetomys ansorgei,* 45.0% identity) (Table J in S1 Appendix). Phylogenetically, these intact mammalian CHIRalphaEnv relatives clustered into distinct rodent (98% bootstrap support) and afrotherian (100% bootstrap support) groups around the chiropteran CHIRalphaEnv lineage (Fig 5). The rodent sequences were nested within the CHIRalphaEnv branch of the phylogeny, suggesting historical transmission between these hosts leading to endogenisation.

Two other mammalian groups also contained degraded copies of CHIRalphaEnv-related sequences: including Sirenia, Proboscidea, Hyracoidea, Macroscelidea, Eulipotyphla and Rodentia. These groups contained CHIRalphaEnv-like sequences with stop codons and/or frameshifts that disrupt the ORF and represent integrations that have not been retained for host function.

Many intact AREs related to CHIRalphaEnv sequences were also present in saurian genomes, notably the brown spotted pit viper (*Protobothrops mucrosquamatus*) (n = 113), the tiger rattlesnake (*Crotalus tigris*) (n = 99) and alligators (*Alligator sinensis* and *A. mississippiensis*) (n = 38, 16 respectively). We also recovered many saurian and testudine sequences within the Mabuya-like clade, indicating these alpharetrovirus envelopes are ubiquitous in reptiles (Fig 5). To explore the evolutionary scenarios that explain this host distribution, we constructed constrained trees in iqTree2 and undertook maximum likelihood topology testing. The null hypothesis, where alpharetrovirus diversity is explained by host codivergence between mammals and reptiles, was rejected with p-values of 0.0005 in the KH and SH tests and 0.000809 in the AU test. The favoured hypothesis was a topology placing mammalian alpharetrovirus sequences nested within the reptilian sequences, indicating at least one cross-class transmission between reptiles and mammals has occurred to explain these observations.

A Bayesian timetree analysis of intact CHIRalphaEnv relatives suggests that the chiropteran CHIRalphaEnv retroviral lineage diverged from saurian CHIRalphaEnv-like viruses approximately 150 million years ago (150.5361 my, 108.74-194.90 95% CI) in the Mesozoic era (S2 Fig). The BEAST analysis also places chiropteran CHIRalphaEnv elements as an ingroup to the other mammalian ERVs, diverging from ERVs in rodents, hyraxes and elephant shrews between 108 and 128 million years ago (77.21-170.96 95% CI, 90% posterior probability) (S2 Fig). A single *Apodemus* ERV is predicted to be closer to chiropteran ERVs than other rodents and may represent another, more recent transmission event ~43 million years ago (S2 Fig).

Retroviruses can recombine and their different genes can often have different evolutionary histories. To also investigate the evolution of the *pol* regions connected to each CHIRalphaEnv ORF we constructed phylogenetic trees of the reverse transcriptase (RT) domain of each *pol* (where available, 6/8 orthogroups)*.* The RT phylogeny reflected that of the CHIRalphaEnv *env* ORF in that chiropteran CHIRalphaEnv were ingroups to both saurian and other mammalian ERVs. The polymerase regions of the other mammalian and saurian CHIRalphaEnv RTs were gammaretroviruses, most similar to ARTgagpol from artiodactyl ERVs [33] (S1 Fig). Simplot recombination analysis confirmed that the polymerase and envelope regions of the CHIRalphaEnv proviruses best matched mammalian gammaretroviruses and avian alpharetroviruses respectively (S8 Fig). This similarity suggests the CHIRalphaEnv envelopes have a long and stable evolutionary association with gammaretrovirus polymerases throughout different host lineages.

## Discussion

We describe an unrecognised ancient lineage of alpharetroviruses that have persisted within Chiroptera for more than 45 million years. This alpharetrovirus lineage (CHIRalphaEnv) arose from a transmission between reptiles and mammals in the Mesozoic period, representing one of the oldest cross-class retroviral transmission events detected. CHIRalphaEnv-like ERVs were also identified in a few other mammals including hyraxes, elephants, manatees, golden moles, elephant shrews and field mice, indicating a far broader host range for alpharetroviruses than has previously been assumed. We show that all eight chiropteran CHIRalphaEnv orthogroups are undergoing purifying selection and propose that these envelopes were acquired and fixed widely amongst chiropterans during a period of rapid diversification to perform a vital host function.

Endogenous retroviruses offer a robust source of information that allows us to identify their time of integration within their vertebrate host. We employed both locus-based orthology determination, Bayesian timetree analysis and 2-LTR dating to estimate the minimum age of CHIRalphaEnv sequences. The youngest sequence in *Rhinolophus* bats integrated around 20 million years ago, whilst the oldest sequences in vespertilionid bats integrated at least (40) million years ago (Table D in S1 Appendix). Some CHIRalphaEnv sequences like CHIRalphaEnv2 and 6 were integrated into highly active genomic regions (containing many transposons), whilst others like CHIRalphaEnv7 seemed to be in much more stable regions (few to no transposons). Remarkably, over these long time periods involving the accumulation of substitutions and transposon activity, the envelope ORFs of each CHIRalphaEnv orthogroup have remained intact and coding competent across bat species. The preservation of an envelope CDS within ERVs is extremely unlikely to occur by chance: only approximately 0.5% of ERV envelopes remain intact under neutral evolution for ~50 million years [40]. Our selection analysis indicates all eight CHIRalphaEnv orthogroups are under purifying selection, suggesting they have been co-opted and maintained convergently in hosts.

Most characterised bat ERVs are relatively young, with ERV integrations concentrated between 10–40 million years ago [19,39,41,42]. With the exception of orthogroup 6 and 7, CHIRalphaEnv elements are predicted to be older than 40 million years (95% confidence interval) and represent some of the oldest cross-family orthologous ERVs identified in bats.

The presence of multiple orthogroups, each unique to a distinct group of chiropterans, indicates the occurrence of multiple co-option events early on during the diversification of Chiroptera 57–63 million years ago [43,44]. The Paleocene/Eocene epochs following the K-Pg extinction ~65 million years ago are characterised by expansion of mammalian lineages associated with a sudden increase in ecological opportunities [45,46]. Rapid host radiation is correlated with increased transposable element activity and ERV invasion, as the decrease of selective pressures can allow genomes to be more tolerant to genetic insertions that cause variation [47,48]. Many retroviral envelopes were co-opted during this period and may have aided the radiation and subsequent specialisation of mammalian lineages by introducing new physiological functions and pathways [26,40,49–51]. Seven of the eight CHIRalphaEnv co-options occurred during the chiropteran radiation in the Eocene epoch and may have similarly contributed to the adaptations and specialisations seen in bat lineages today [43,44,52].

Envelope co-option has been utilised for a myriad of functions within vertebrates, most notably for antiviral defence or development of the placenta. The observed prevalence of CHIRalphaEnv transcripts within endometrium samples and low expression in all other tissues would suggest the element has a role in placentation or reproduction. However, this hypothesis cannot explain the heightened transcription detected in foot samples from a *Myotis myotis* bat. This bat was infected with the fungus *Pseudogymnoascus destructans* which causes white-nose syndrome (WNS), a potent pathogen in hibernating bats. *Myotis myotis* show little transcriptional dysregulation during WNS infection [53], however it is possible that CHIRalphaEnv is activated as an immunomodulatory protein in response. However, at least 51 of the RNA-Seq experiments were from bats with WNS and only 12 contained detectable levels of CHIRalphaEnv expression (Table H in S1 Appendix), indicating that CHIRalphaEnv expression is not reliably correlated with this disease.

Similarly, a further 47 RNA-Seq experiments were from bats infected with various viruses, of which only six displayed CHIRalphaEnv expression. The lack of expression in response to infection and absence in immune tissues (e.g., Spleen, lymph nodes) precludes the antiviral function of CHIRalphaEnv based on current knowledge [54,55]. We therefore focus on the possible reproductive role of CHIRalphaEnv elements based on endometrial expression.

The CHIRalphaEnv orthogroups have features in common with syncytin genes, retaining intact ORFs and demonstrating high endometrium-specific expression (Fig 4). To be designated as a *bona fide* syncytin, ERV envelopes generally fit three criteria: display placenta-specific expression, display fusogenic properties and be evolutionary conserved over extended periods of evolution [23]. The absence of fusogenic activity in bat, rodent and human cell lines indicates that CHIRalphaEnv elements are likely performing a non-fusogenic physiological role in bat reproduction rather than functioning as a canonical syncytin. Notably, not all syncytins have fusogenic activity, some have lost their fusogenicity but retain placenta-specific expression and may still play a role in maternal immunotolerance [50,56]. For example, *envV* in humans retains all three hallmarks in Old World monkeys but has lost fusogenic activity in New World primates [26] and *env-Can1* in tenrecs similarly shows high expression in the placenta but no fusogenic activity [57]. In humans and mice syncytins are conserved in pairs, one of which contributes to immunotolerance and the other is responsible for cell-cell fusion [56]. In addition, some co-opted envelopes have been evolutionarily conserved for no clear function, including *pan-Mars-env2* in marsupials and HEMO (MER34) family proteins in primates and felines [58,59]. Our observations of ubiquitous and preserved CHIRalphaEnv ERVs that are highly expressed in reproductive tissue supports the hypothesis that these elements complemented the ancestral mammalian syncytins in chiropterans. Further exploration of CHIRalphaEnv expression as bat transcriptomes and proteomes become available, particularly from reproductive organs, is vital to understanding the function of these elements across Chiropterans. Unlike previously characterized syncytins, CHIRalphaEnv are the first mammalian syncytin candidates related to alpharetroviruses. The best characterised syncytins, syncytin-1 and -2 in humans and syncytin-A and -B in mice are derived from gammaretroviral envelopes, specifically ones similar to type-D recombinant betaretrovirus envelopes (Fig 1) [60]. Logically, searches for syncytins in other mammals used these elements as a reference, resulting in the discovery of other gammaretroviral syncytins like Car1 and Rum1 [49,61]. In contrast, CHIRalphaEnv envelopes are phylogenetically closer to *Alpharetrovirus* than *Gammaretrovirus*, containing many motifs and hallmarks unique to avian retroviruses (Figs 1 and 3). This finding supports the hypothesis that syncytins are not only gammaretrovirus-derived and instead are driven by the versatility of retroviral envelopes from all taxa. Our results highlight alpharetroviruses as an underappreciated source of exapted endogenous retroviral envelopes that contribute to host biology.

Due to the limited sample sizes for some tissues (e.g., Only two samples for both foot and endometrium), unambiguously suggesting a function to CHIRalphaEnv elements is challenging. As more RNA sequencing and proteomes from different tissues and bat species become available, it will be useful to explore CHIRalphaEnv transcription in the context of broader physiological roles.

The closest previously described CHIRalphaEnv relatives were in saurian (*Mabuya* lizard) and amphibian (caecilian) genomes [17,62]. The CHIRalphaEnv elements discovered in this study form a sister lineage to the *Mabuya* and caecilian elements, suggesting there are several either unsampled or extinct lineages of alpharetroviruses infecting other tetrapods. The presence of degraded CHIRalphaEnv copies in chiropteran genomes also indicates that the CHIRalphaEnv virus was highly successful and prominently infected bats throughout their evolution. Although no extant bat retroviruses with alpharetrovirus envelopes have been isolated, it would be useful to determine if this lineage is still present in bats or has since become extinct. Taken together, this indicates that alpharetrovirus envelopes have ancient origins and a much wider host range than previously appreciated.

The phylogenetic placement of the CHIRalphaEnv lineage, nested within a clade of saurian, crocodilian and amphibian retroviruses, strongly supports an origin outside mammals, and that at least one cross-class transmission event occurred. All CHIRalphaEnv orthogroups were recombinant retroviruses, with *env* sequences of alpharetroviral origin and *gag*/*pol* regions more closely related to the gammaretroviruses (n = 7) or ERV-L retroviruses (n = 1).

The phylogenetic congruence between the *pol* and *env* trees of CHIRalphaEnv orthogroups 2–8 supports transmission of the linked *pol*-*env* genes of an ancestral recombinant retrovirus was transmitted between classes rather than a *pol*-*env* recombination event during the saurian-mammal jump. In contrast, CHIRalphaEnv1 had a very different polymerase from the other CHIRalphaEnv elements, indicating at least one recombination event occurred in the history of this viral lineage.

CHIRalphaEnv sequences were present in few other mammals, suggesting their host range was limited after transmission, or their genomic persistence was highly variable. Three superorders of mammals contained CHIRalphaEnv-like ERVs: Laurasiatheria (bats, moles), Afrotheria (dugongs, elephants, hyraxes, elephant shrews) and Euarchontoglires (rodents) (Fig 5 and Table J in S1 Appendix). Evolutionarily, this implies one of two scenarios: [1] CHIRalphaEnv viruses were introduced to the common ancestor of these three mammalian superorders, in which case we would expect to find CHIRalphaEnv relatives in other mammals including Carnivora and *Mus*, or [2] distinct transmission events gave rise to CHIRalphaEnv groups in each mammalian lineage. Our observations support scenario two given the lineage-specific detection of CHIRalphaEnv elements in distantly related hosts. It is, however, still possible that the ancestral mammal was infected, and only some mammalian lineages were tolerant to ERV integration or preserved the ERV well enough for detection using our methods. Further ERV-mining as more mammalian and saurian genomes become available will help reconstruct the probable scenarios surrounding this cross-class transmission.

The acquisition of CHIRalphaEnv independently by different bat families at different evolutionary points may contribute to the remarkable variety of structural and physiological strategies during bat reproduction [63]. The acquisition of new syncytins can affect placenta biogenesis and maternal immunotolerance, allowing the transition between placentation types, for example from hemochorial to endotheliochorial placentation in ruminants [61]. Intriguingly, aside from primates, bats are one of the only mammals to display hemochorial placentation. Hemochorial placentation poses many physiological challenges due to the lack of maternal separation from the embryo, and immune-regulated genes are widely upregulated at the maternal-foetal interface to compensate [64]. The conservation of the ISD within all CHIRalphaEnv orthogroups and heightened expression in the endometrium supports an immunosuppresive function during pregnancy.

Beyond roles in immunotolerance during pregnancy, the taxonomic distribution of CHIRalphaEnv is suggestive of a possible role in the physiology of endometrial shedding required for “true” menstruation. Bats are one of few mammals aside from primates that exhibit “true” menstruation, defined as spontaneous endometrial decidualization wherein the endometrium is shed in the absence of foetal implantation [65]. This spontaneous decidualization is thought to be partly triggered by immunostimulation in the endometrium [66], a process to which expression of retroviral envelopes could contribute through the stimulation of pattern recognition receptors [67,68]. Aside from bats and primates, true menstruation is also documented in the afrotherian elephant shrews (*Elephantulus*) and rodent spiny mice (*Acomys*) [65], both of which also have CHIRalphaEnv-like elements. This suggests the possibility that CHIRalphaEnv and related envelopes have promoted the development of menstruation in these mammalian lineages. Further studies exploring CHIRalphaEnv expression throughout the menstrual cycles of bats, afrotherians and Murinae rodents will help elucidate if they contribute to this unique physiology within these groups.

Overall, we provide evidence of a novel lineage of alpharetroviruses that have influenced bat evolution over millions of years. These CHIRalphaEnv elements provide an opportunity to explore retroviral transmission dynamics in the Mesozoic era, having originated from saurian hosts and transmitted into at least three different mammalian lineages. The repeated functional co-option of this lineage provides a unique opportunity to study the co-option of retroviral envelopes for host physiology: within Chiroptera there have been at least eight co-option events of CHIRalphaEnv elements. The ancient origins, repeated co-option and broad host distribution of this lineage illustrates the dynamic interplay between retroviruses and their hosts and provides a model of how endogenous retroviruses can be used as both a molecular fossil record and tool to explore host biology and ecology.

## Methods

### Identification of alpha-like envelope elements in host genomes

**Fig 6:**
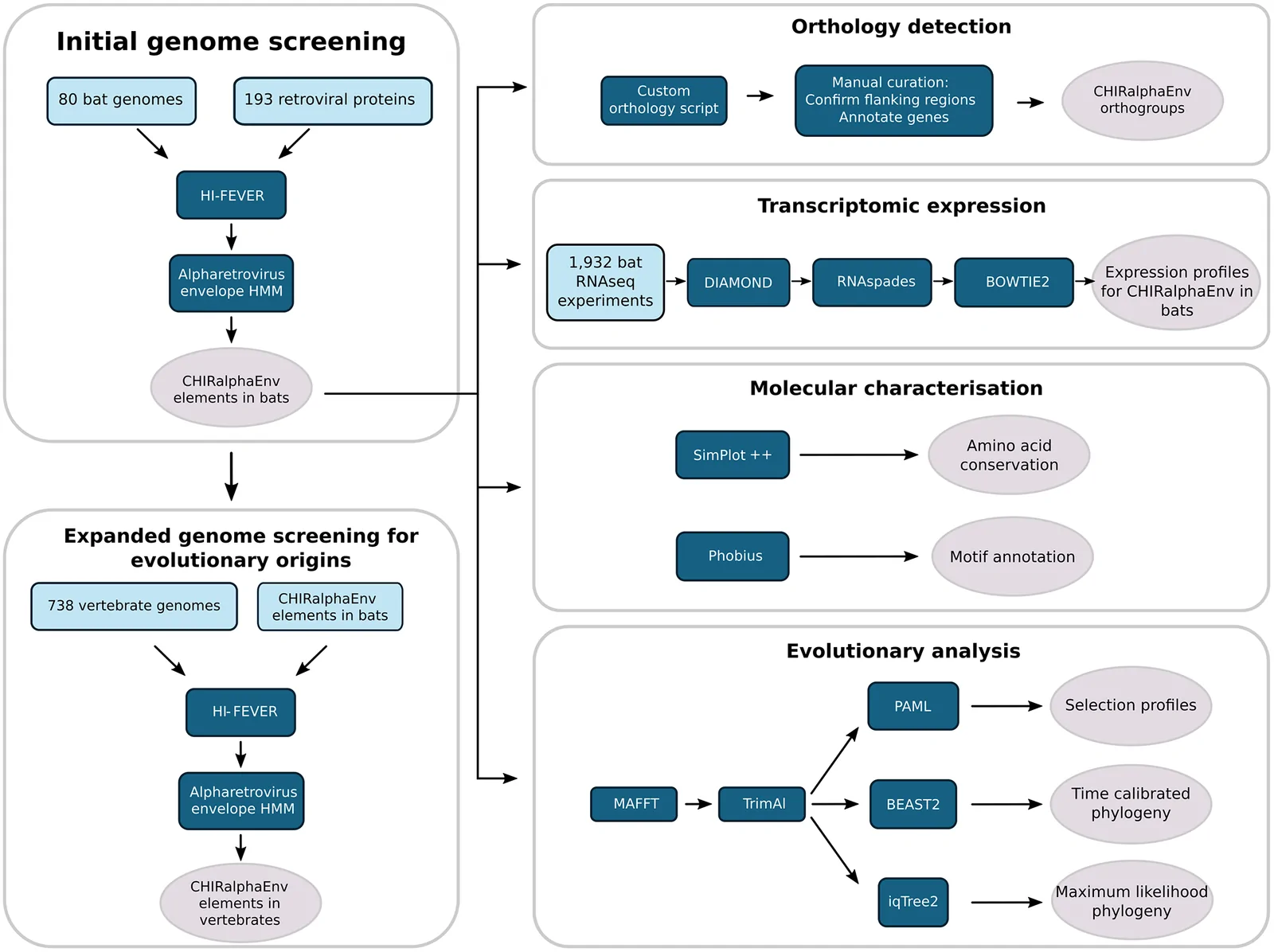
Workflow used to identify, classify and characterise CHIRalphaEnv elements.

We screened vertebrate genomes for ERVs with alpha-like envelopes using HI-FEVER: an EVE-discovery workflow [31] (Fig 6). Retroviral proteins (n = 193) spanning all extant and endogenous retroviral genera were used to query the bat genomes (n = 80) using a DIAMOND [69] search implemented in HI-FEVER. Alpha-like sequences identified from this search were used to query other vertebrate genomes (n = 738,Table I in S1 Appendix).

ERVs can cross-match with retrotransposons and other viral glycoproteins in similarity searches [70,71]. We therefore implemented an additional confirmation step to distinguish alpharetrovirus-like envelopes. A custom Hidden Markov Model (HMM) was trained on alpharetrovirus envelope sequences (n = 6) using the HMMER tool (http://hmmer.org) and used to identify alpha-like envelopes in the HI-FEVER output. ERVs that matched the alpharetrovirus HMM profile (E-value < 1e^-10^) and retained an intact ORF of >300 aa were selected for further investigation.

### Orthology determination

The identification of orthologous ERVs in two or more species indicates that the ERV integrated before the divergence of the host lineages. To determine if any CHIRalphaEnv sequences were orthologues, we compared flanking host genomic sequences (30 kB) and assessed their similarity using previously defined methods [70] (Fig 6). Briefly, ERV flanks were compared all-against-all in a local BLASTn search, and hits with coverage >70% were considered significant. The nucleotide identity of these hits was used to determine matches and populate an adjacency matrix by defining an edge between sequences when the sum of their flank matches was greater than 1. A graph was then created from this adjacency matrix using the NetworkX python library and connected components were extracted as orthogroups.

As a second, independent test of orthology, proximal host genes upstream and downstream of the integrated ERVs were identified using NCBI genome data viewer on well-annotated bat genomes. The protein sequences of these proximal genes were used for tBLASTn searches to other, less annotated, bat genomes and cross-referenced with the CHIRalphaEnv loci. Relative positions of host gene matches and absolute distance from the insertions was used to confirm orthology between bat genomes.

Given that the elements of each orthogroup share the same canonical motifs and sequence structure, we randomly selected the representative sequences for use in further analyses, where applicable (Table D in S1 Appendix).

### Characterisation of flanking retroviral genes and proviruses

As retroviral envelopes are often part of a larger proviral integration, we identified remnants of other retroviral features surrounding the envelope CDS. To identify other retroviral core genes (*gag/pol*), 10 kB flanking regions were extracted and queried using a DIAMOND search with the initial library of 193 retroviral protein queries. Resulting hits were classified by combining results from reciprocal DIAMOND searches of the NCBI non-redundant database and reference viral database (RVDB), and through an HMMER search against the Gag and Pol HMMs from GyDB [72].

Full proviruses are flanked by two long terminal repeats (LTRs) which define the boundaries of the ERV element. To identify long terminal repeats, the envelope CDS plus 20 kB flanks were given to LTR harvest [73] as inputs. Short LTR sequences (<300 nt) were extended (where possible) using BLASTn to identify regions of identity flanking each provirus. The retroviral origin and identity of LTR candidates was confirmed using a search of the Censor GIRI database [35].

### Envelope characterisation

We used Phobius (https://phobius.sbc.su.se/) to identify signal peptides and transmembrane domains as these are hallmark features of retroviral envelope proteins [74]. The furin cleavage site, fusion peptide and immunosuppressive domain (ISD) were manually identified based on previously characterized retrovirus envelope primary sequence motifs in the literature.

To identify regions of shared nucleotide identity between orthogroups within the envelope ORF, we aligned the ORFs using MAFFT and used SimPlot++ [75] with a Jukes-Cantor nucleotide substitution model and 200 bp sliding windows with 20 bp steps to visualize the identity across the ORF. Orthogroup 1 was used as the reference for comparison as it was the most divergent from the other orthogroups.

### Selection analysis

To determine whether sequences showed signals of selection we used PAML v4.10.7 [76] (Fig 6). Models M0, M1a, M2a, M7 and M8 with the F1X4 codon substitution model were used to predict purifying and positive selection on the whole tree with one ω (dN/dS) for all branches. The significance of each comparison (test statistic) was calculated using 2Δl where Δl is the difference in log-likelihoods between models. Significance was assessed by comparing the test statistic (2Δl) at the level of p = 0.05 and the difference in the degrees of freedom, to the Chi-squared distribution.

To identify signals of selection among individual orthogroups we iterated through each orthogroup as a foreground branch and ran the M2a model with site model 2 allowing for one or more dN/dS ratios for branches.

### Transcriptomic analysis

To investigate if any chiropteran alpharetrovirus envelope (CHIRalphaEnv) sequences were transcribed in bat cells, we conducted DIAMOND searches querying the raw reads of SRA experiments (Table H in S1 Appendix) against CHIRalphaEnv sequences (Fig 6). SRA experiments were considered to have positive expression if they contained >5 reads with 100% identity over their full length to any of the CHIRalphaEnv sequences. For SRA experiments with CHIRalphaEnv expression, reads were normalized using the housekeeping gene *EEF1A1* [77] with the following formula: Normalized reads = raw reads * (housekeeping reads / total reads).

SRA experiments with >1000 CHIRalphaEnv reads were *de novo* assembled into transcripts using RNASpades [78]. A tBLASTn search with the CHIRalphaEnv peptide sequence was used to identify CHIRalphaEnv transcripts from the pool of assembled transcripts. Raw RNA-Seq reads were mapped to CHIRalphaEnv transcripts using BOWTIE2 and read coverage was calculated using SAMTOOLS [79,80].

### Phylogenetic analysis and time-calibrated tree

To determine the evolutionary relationships between CHIRalphaEnv elements, peptide sequences were aligned using MAFFT and trimmed using TRIMal (-gappyout) to remove poorly-aligned regions [81]. Maximum-likelihood phylogenetic trees were constructed using IQTREE2 (JTT + R5 substitution model) with 1000 ultrafast bootstraps and outgroup rooted [82] (Fig 6).

Bat divergence times were derived from timetree.org [83] which compiled information from multiple studies to provide median divergence times and confidence intervals which were used for prior calibrations of the BEAST2 timetree. The divergence times and confidence intervals compiled by timetree are consistent with previous estimates of mammalian evolution [84,85]. The tree used and estimated divergence times are available as S9 Fig and in Table D in S1 Appendix respectively.

A time-calibrated tree of CHIRalphaEnv elements was inferred in BEAST2 using model JTT + I + G4 and an optimized relaxed clock [86]. Internal chiropteran nodes were calibrated with MRCA priors using a log normal (LN) or normal (N) distribution to best match the estimated divergence times and confidence intervals from Timetree.org [83] (S9 Fig) as follows: CHIRalphaEnv1: N 35my (8.4-39 CI), CHIRalphaEnv2: LN 45my (38.7-48.1 CI), CHIRalphaEnv3: LN 42my (40.7-45.7 CI), CHIRalphaEnv4: LN 45my (38.7-48.1 CI), CHIRalphaEnv5: LN 42my (40.7-45.7 CI), CHIRalphaEnv6: LN 19.1my (18–21 CI), CHIRalphaEnv7: LN 29.7my (25.1-32.8) and CHIRalphaEnv8: LN 41my (39-47.5 CI). The analysis was run with a Markov Chain Monte Carlo (MCMC) length of 100 million generations and inspected in Tracer to confirm good mixing, stationarity and an effective sample size >200 for all parameters. The posterior tree sample was summarized as a maximum clade credibility tree using TreeAnnotator applying 25% burn-in.

Topology testing of evolutionary hypotheses was conducted in IQTREE2. A constrained tree was generated placing the mammalian alpharetrovirus-like sequences as a monophyletic sister group to alpharetroviruses, with gammaretroviruses as an outgroup. The log-likelihood of this tree was compared with that of an unconstrained tree using IQTREE2 with the RELL approximation and 10,000 replicates.

In BEAST, the same alignment was used to construct a Bayesian tree using model JTT + G4 with estimated invariable site proportions and gamma shape and an optimized relaxed clock and a Yule model.

### Cells and plasmids

Lenti-X 293T cells (Takara Bio, Cat. #632180), Pakit03 (*Pteropus alecto*, Accession: CVCL_DR89), TB1.Lu (*Tadarida brasiliensis*, Accession: CVCL_2730) MDTF (*Mus dunni*, Accession:CVCL_J394) and HT1080 (*Homo sapiens*, Accession: CVCL_0317) were cultured in complete growth medium: Dulbecco’s Modified Eagle Medium (DMEM) high glucose (Sigma-Aldrich, D6429), supplemented with 10% fetal bovine serum (FBS) (Gibco, A5256801), 100 U/mL penicillin–streptomycin (Sigma-Aldrich, P4333), and 100 μg/ml Normocin (InvivoGen, ant-nr-2), at 37°C with 5% CO_2_.

For the positive control plasmid, a truncated MLV-env with the 16nt p2E cytoplasmic tail removed [87,88] and a Kozak sequence (GCCACC) added was synthesised by Twist Bioscience and cloned into pTwist ENTR. This plasmid was then recombined into the pcDNA3.1(+) GatewayIRESmScarlet expression plasmid using Gateway LR Clonase II Enzyme Mix (Invitrogen, 11791020) to get pcDNA3.1(+)-MLV-env-IRESmscarlet. pcDNA3.1(+) GatewayIRESmScarlet was generated from pcDNA3.1(+) by insertion of GatewayIRESmScarlet.

CHIRalphaEnv element genes containing a Kozak sequence (GCCACC) were synthesized by Twist Bioscience and cloned into pTwist ENTR and entry clone into the pLGatewayIRESmScarlet expression plasmid with Gateway LR Clonase II Enzyme Mix (Invitrogen, 11791020). The pLGatewayIRESmScarlet backbone was derived from pLGatewayIRESeYFP [89] by replacing eYFP with mScarlet. The Mo-MLV Gag-Pol vector (KB4) [90] and pVSV-G (Addgene #8454) were used as the packaging and envelope plasmids for virus production.

### CHIRalphaEnv production

Viruses were produced by transfecting Lenti-X 293T cells with a three-plasmid system consisting of pVSV-G, KB4, and pLGatewayIRESmScarlet carrying the CHIRalphaEnv elements by using GenJet In Vitro DNA Transfection Reagent (Ver. II) (SignaGen, #SL100489) according to the manufacturer’s instructions. At 12 h post-transfection, cells were treated with 10 mM sodium butyrate for 8 h [91]. Virus-containing supernatants were harvested at 72 h post-transfection, filtered through a 0.45 µm PES filter (Fisherbrand, 15216869), aliquoted, labelled, and stored at −70°C. Viral titres were subsequently determined by FACS [92].

### Transduction and assessment of syncytia formation

For CHIRalphaEnv elements, Pakit03, Tb1.Lu, MDTF and HT1080 cells were transduced with the collected virus-containing supernatants. Briefly, 2.5 × 10^4^ cells per well were seeded into 12-well plates and incubated overnight. On the following day, cells were counted and transduced with virus-containing supernatant at an MOI of 0.5 in complete growth medium supplemented with 8 µg/mL polybrene (Sigma-Aldrich, TR-1003) [93]. mScarlet reporter expression was assessed at 24 h post-transduction to confirm successful transduction.

For the pH4 acid-treated group, 1 mL of pH4 Dulbecco’s phosphate-buffered saline (DPBS) (Gibco, 14190144; adjusted with 37% hydrochloric acid, 320331–500ML) was added for 5 min at 36 h post-transduction [17]. Cells were then returned to complete growth medium and incubated for an additional 12 hours.

For positive syncytia controls, Pakit03 and MDTF cells were seeded into 12-well plates and incubated until 70 ~ 80% confluent. pcDNA3.1(+)-MLV-env-IRESmscarlet was transfected into cells using GenJet In Vitro DNA Transfection Reagent (Ver. II) (SignaGen, #SL100489) according to the manufacturer’s instructions. Phenotypes were assessed at 36 h post-transduction to confirm successful transduction.

Before syncytia formation analysis, cells were stained for 10 minutes in the dark with Hanks’ Balanced Salt Solution (HBSS) (Gibco, 14025092) containing 5 µg/mL Hoechst 33342 (TargetMol Chemicals Inc., T5840) and 5 µg/mL Lectin from Triticum vulgaris (wheat, FITC conjugate) (Sigma-Aldrich, L4895). Images were captured with EVOS FLoid Cell Imaging Station (Life Technologies).

Microscopy images were edited with GNU Image Manipulation Program (GIMP, version 3.2.0) to adjust the brightness, contrast and opacity of each layer. For the red and green layers, the background was removed by firstly reducing the image to two colours using the ‘threshold’ function and using the ‘color to alpha’ function to remove the background. For the green layer, the dye was enhanced using the ‘High pass’ and ‘Noise reduction’ filters. Layers were overlaid onto the white light image to create the final composites. All original images are provided on the OSF repository.

## Supporting information

S1 Fig Maximum likelihood tree of CHIRalphaEnv polymerase sequences. Sequences were aligned with MAFFT, trimmed with TrimAL and a phylogeny was generated with iqTree2 with 1000 Ultrafast bootstrap replicates.
(TIFF)

S2 Fig Bayesian timetree of CHIRalphaEnv and related elements in vertebrates. Purple bars represent the 95% confidence intervals of estimated divergence times. Branches are labelled with posterior probabilities.
(PDF)

S3 Fig CHIRalpha orthogroup 2 syncytia experimental results. Pink cells denote mScarlet and CHIRalphaEnv expression, blue represents cell nuclei and green represent cell membranes.
(TIFF)

S4 Fig CHIRalpha orthogroup 3 syncytia experimental results. Pink cells denote mScarlet and CHIRalphaEnv expression, blue represents cell nuclei and green represent cell membranes.
(TIFF)

S5 Fig CHIRalpha orthogroup 4 syncytia experimental results. Pink cells denote mScarlet and CHIRalphaEnv expression, blue represents cell nuclei and green represent cell membranes.
(TIFF)

S6 Fig CHIRalpha orthogroup 5 syncytia experimental results. Pink cells denote mScarlet and CHIRalphaEnv expression, blue represents cell nuclei and green represent cell membranes.
(TIFF)

S7 Fig CHIRalpha orthogroup 7 syncytia experimental results. Pink cells denote mScarlet and CHIRalphaEnv expression, blue represents cell nuclei and green represent cell membranes.
(TIFF)

S8 Fig Recombination analysis of CHIRalphaEnv elements compared to gammaretrovirus and alpharetrovirus references. Two alpharetroviruses (NC_001407.1 and BK071386.1) and two gammaretroviruses (NC_039228.1 and BK068965.1) were used as references against the query of all CHIRalphaEnv orthogroup representative sequences. The alignment spans the pol (1–3,200 nt) and env (3,104–5,300 nt) regions of the viruses.
(TIFF)

S9 Fig Chiropteran timetree with predicted CHIRalphaEnv integration times. Timetree was generated with Timetree.org [83] showing host divergence times, geological timescale, earth impacts, oxygen and carbon dioxide levels and solar luminosity. CHIRalphaEnv integration times were predicted based on orthology and host divergence dates and plotted on the tree. Colours correspond with those in Fig 2: light green = CHIRalphaEnv8, dark green = CHIRalphaEnv1, khaki = CHIRalphaEnv7, dark blue = CHIRalphaEnv5, maroon = CHIRalphaEnv3, red = CHIRalphaEnv2, blue = CHIRalphaEnv4.
(TIFF)

S1 Appendix Table A. Chiropteran genomes screened and Alpha-like retroviral envelopes (AREs) detected. Table B. Total CHIRalphaEnv elements in chiropterans (BLAST results). Table C. Intact CHIRalphaEnv elements in chiropterans. Table D. CHIRalphaEnv orthogroup summaries. Table E. Polymerase BLAST results for CHIRalphaEnv proviruses. Table F. LTR dating of CHIRalphaEnv orthogroup 7. Table G. PAML selection analysis results. Table H. SRA experiments screened for CHIRalphaEnv sequences. Table I. Non-chiropteran vertebrate genomes screened for CHIRalphaEnv sequences. Table J. CHIRalphaEnv-like elements in vertebrates.
(XLSX)
