# Supplementary material for: An ancient alpharetrovirus lineage in bats: Evolutionary insights and possible roles in reproduction
Source: PLoS Genet. 2026 Aug 25;22(8):e1012277. doi: 10.1371/journal.pgen.1012277 (PMC13568524; doi:10.1371/journal.pgen.1012277)
Supplement: Supplementary file 1 [file pgen.1012277.s002.pdf]

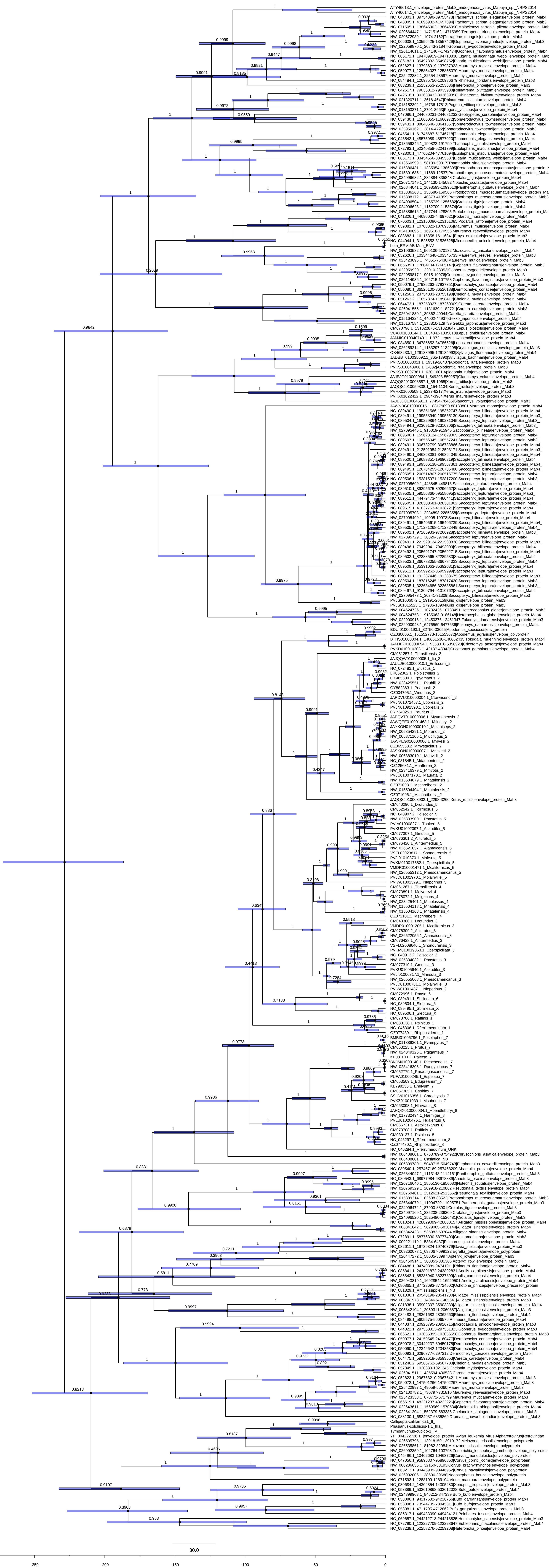
30.0
-250
-200
-150
-100
-50
0
