# Supplementary material for: An ancient alpharetrovirus lineage in bats: Evolutionary insights and possible roles in reproduction
Source: PLoS Genet. 2026 Aug 25;22(8):e1012277. doi: 10.1371/journal.pgen.1012277 (PMC13568524; doi:10.1371/journal.pgen.1012277)
Supplement: Supplementary file 2 [file pgen.1012277.s012.pdf]

Response to editors

Dear editorial team of PLoS genetics.

Thank you for your consideration of our article "An ancient alpharetrovirus lineage in bats: evolutionary insights and possible roles in reproduction". We welcome your comments and have addressed them below in detail. Briefly, we have modified language referring to CHIRalphaEnv as a syncytin candidate, instead emphasising its co-option and conservation. We have clarified several claims to correctly represent the results from this study and added an author summary to the text.

1. Reconcile the syncytin framing with your own negative fusogenicity result. *This is the most important point. Your new experiments found no syncytia across the four cell lines tested, and you now correctly describe the elements as "non-fusogenic" in the Results and in your cover letter. However, the manuscript continues to advance canonical syncytin language elsewhere: the Abstract proposes "the first mammalian syncytin candidate," the Discussion (around line 503) states that "CHIRalphaEnv are the first mammalian syncytin candidates related to alpharetroviruses," and "syncytin" remains a listed keyword. A non-fusogenic element does not meet the standard functional definition of a syncytin, so as written, these statements are internally inconsistent with the data.*

*Please resolve this throughout the manuscript by either (a) replacing "syncytin candidate" with more accurate language, such as "co-opted/exapted envelope with a likely reproductive role," or (b) if you wish to retain a syncytin-related framing, explicitly defining a non-fusogenic, syncytin-like category (**note that I do not favor this unless you can appropriately justify this; the two mouse syncytin examples you cite are less clear about this dichotomy**) and justifying it against the conventional fusogenicity criterion, with the negative assay foregrounded rather than downstream. The hedged title ("possible roles in reproduction") is appropriate and may stand; the Abstract and Discussion need to be brought into line with it.*

We have amended the wording alluding to the predicted function of CHIRalphaEnv and removed references to it being a syncytin. We have also removed syncytin from the keyword list for the article. The text now reads:

"We also propose the first mammalian <u>co-opted</u> E<u>RV</u> derived from an *Alpharetrovirus* envelope"

We have also moved the experimental results earlier in the text to preface the discussion around alternative non-canonical syncytin activity. The text now reads:

“The absence of fusogenic activity in bat, rodent and human cell lines indicates that CHIRalphaEnv elements are likely performing a non-fusogenic physiological role in bat reproduction rather than functioning as a canonical syncytin. Notably, not all syncytins have fusogenic activity, some have lost their fusogenicity but retain placenta-specific expression...”

2. Make the genome and element counts consistent across the entire manuscript. *The methodological overhaul has left the numbers unreconciled. The number of genomes screened appears variously as 78, 79, and 80 (e.g., "n=79" in the revised Methods versus "80 available chiropteran genomes" at Results line 112). The number of genomes with detections appears as both "all 79 genomes" and "73 genomes" (Results line 115). The count of intact >300-aa elements appears as 141 (Results line 115), 142 (elsewhere in the Results and in your responses), and 94 (legacy figure). Please choose the correct, current values and apply them uniformly across the Abstract, Results, Methods, all figures, and the supplementary tables, and confirm that "detected in all genomes" versus "detected in 73 genomes" is stated consistently.*

We have thoroughly reviewed the numbers in the manuscript, figures and tables for consistency. We now list screening 80 bat genomes, of which 79 had CHIRalphaEnv elements and 73 had intact ones >300 AA. Amended text is listed below:

“AREs were detected in 79/80 genomes comprising 2,739 total sequences of which 141 were intact”

3. Verify every figure and supplementary table cross-reference. *Several supplementary tables were renumbered during revision (e.g., the reviewer-flagged corrections of Table 5→8 and Table 6→7). Some in-text citations still appear to point to pre-renumbering targets. Please perform a complete pass confirming that every Figure and Supplementary Table/Figure callout in the text resolves to the intended item, paying particular attention to the positive-selection site table and the RNA-seq/expression table.*

We have reviewed the figure and supplementary table cross-references and renumbered where required.

4. Make the underlying data publicly accessible and provide a DOI. *The data are currently shared via an anonymized, view-only OSF link. PLOS policy requires that the repository be publicly accessible at acceptance and cited by a persistent identifier. Please make the OSF project public, provide its DOI in the Data Availability Statement,*

and confirm that the alignments and tree files underlying Figures 1, 2, and 5 and Supplementary Figure 1 are all included, as committed in your responses.

We have made the OSF repository associated with this study public. It can be found at https://osf.io/jc2g4. We have confirmed that all cited files are uploaded on the repository and have amended the text to reflect the new link (below):

"CHIRalphaEnv sequences, alignments, phylogenetic trees, HMM profile and microscopy images generated in this study are available on the Open Science Framework repository (https://osf.io/jc2g4)."

5. Calibrate two interpretive claims to the strength of the evidence. *These do not require new analysis, only adjusted wording. (i) The proposed expansion of the alpharetrovirus host range into Afrotheria and rodents rests on BLASTx similarity (~45% amino-acid identity) from a single CHIRalphaEnv7 query; please ensure the Abstract's claim of expansion "into multiple mammalian clades" is phrased as a similarity-based inference rather than a demonstrated finding. (ii) The reproductive-function argument rests on two endometrium samples; your added limitation text is appropriate, and I would simply ask that the Abstract's "indicating an involvement in reproductive function" be softened (e.g., "suggesting") to match.*

We have amended the text as suggested. It now reads:

"We observe high expression levels of CHIRalphaEnv envelopes in endometrium tissue from *Carollia perspicillata,* suggesting an involvement in reproductive function. We find CHIRalphaEnv sequence relatives in multiple mammalian clades (Afrotherians, rodents and bats), expanding the host range and extending origins of alpharetroviruses beyond 43 million years."
