# Supplementary material for: An ancient alpharetrovirus lineage in bats: Evolutionary insights and possible roles in reproduction
Source: PLoS Genet. 2026 Aug 25;22(8):e1012277. doi: 10.1371/journal.pgen.1012277 (PMC13568524; doi:10.1371/journal.pgen.1012277)
Supplement: Supplementary file 3 [file pgen.1012277.s011.docx]

**Response to Reviewers**

*Formatting – reviewer comments are in black, author responses are in grey and amended text from the manuscript is in blue.*

## Reviewer 1

In this manuscript, Harding et al. described a lineage of alpharetrovirus-like envelopes in bats. They identified alpharetrovirus-like ENVs in all 78 chiropteran genomes. These CHIRalpha elements are highly expressed in embryonic cells. This is an expansion of their previous finding (PMID: 32699395). More wet lab work is needed to improve its novelty.

1. The RT phylogeny shows that CHIRalpha RTs are closely related to gammaretroviruses. More evolutionary concordance is needed.

We thank the reviewer for their suggestion and have conducted additional analysis to support the recombinant nature of the CHIRalphaEnv viral lineage. To demonstrate the discordance between the gag/pol regions and the env region we used the Simplot software to compare the nucleotide identity of the proviruses against alpharetrovirus and gammaretrovirus reference sequences. The Simplot graph is available as Supplementary Figure 8. We have also added the following text to the results section describing this:

“Simplot recombination analysis confirmed that the polymerase and envelope regions of the CHIRalphaEnv proviruses best matched mammalian gammaretroviruses and avian alpharetroviruses respectively (Supplementary Figure 8).”

We also added a note regarding the different recombinant nature of CHIRalphaEnv1, indicating that the CHIRalphaEnv has affiliated with at least two distinct pol lineages over time.

“In contrast, CHIRalphaEnv1 had a very different polymerase from the other CHIRalphaEnv elements, indicating at least one recombination event occurred in the history of this viral lineage.”

2. To claim syncytin function, inducing the formation of syncytia is needed. This is a simple experiment.

We thank the reviewer for this suggestion and have included syncytia experiments. We tested four cell lines (rodent, human, *Yangochiroptera*, *Yinpterochiroptera*) for fusogenic activity after transduction of CHIRalphaEnv elements into the cells. The methods have been added (lines 712-772). We did not observe any syncytia formation in these cells and have presented this in the results:

“To explore the possible fusogenic function of CHIRalphaEnv elements, we expressed CHIRalphaEnv orthogroups 2, 3, 4, 5 and 7 in human, bat and rodent cell lines under both normal conditions and after acid treatment (pH4). No synctyia were observed under these conditions, concordant with a loss of fusogenic activity (Supplementary Figures 3-7).”

Based on these findings, we have changed the emphasis of the discussion from CHIRalphaEnv being a canonical fusogenic syncytin to being a non-fusogenic element involved in reproduction or development. The amended discussion now reads:

“In humans and mice syncytins are conserved in pairs, one of which contributes to immunotolerance and the other is responsible for cell-cell fusion.”

“The absence of fusogenic activity in bat, rodent and human cell lines indicates that CHIRalphaEnv elements are likely performing a non-fusogenic physiological role in bat reproduction.”

We have also added discussion around alternate hypotheses for CHIRalphaEnv function. The new text from the discussion now reads:

“Envelope co-option has been utilised for a myriad of functions within vertebrates, most notably for antiviral defence or development of the placenta. The observed prevalence of CHIRalphaEnv transcripts within endometrium samples and low expression in all other tissues would suggest the element has a role in placentation or development. However, this hypothesis cannot explain the heightened transcription detected in foot samples from a Myotis myotis bat. This bat was infected with the fungus Pseudogymnoascus destructans which causes white-nose syndrome (WNS), a potent pathogen in hibernating bats. Myotis myotis show little transcriptional dysregulation during WNS infection (49), however it is possible that CHIRalphaEnv is activated as an immunomodulatory protein in response. However, at least 51 of the RNA-Seq experiments were from bats with WNS and only 12 contained detectable levels of CHIRalphaEnv expression (Supplementary Table 8), indicating that CHIRalphaEnv expression is not reliably correlated with this disease.

Similarly, a further 47 RNA-Seq experiments were from bats infected with various viruses, of which only six displayed CHIRalphaEnv expression. The lack of expression in response to infection and absence in immune tissues (eg. Spleen, lymph nodes) precludes the antiviral function of CHIRalphaEnv based on current knowledge (50, 51). We therefore focus on the possible developmental role of CHIRalphaEnv elements based on endometrial expression.”

Finally, we also added text highlighting the limitations of any hypotheses built upon small sample sizes. The added discussion text reads:

“Due to the limited sample sizes for some tissues (eg. Only two samples for both foot and endometrium), unambiguously suggesting a function to CHIRalphaEnv elements is challenging. As more RNA sequencing and proteomes from different tissues and bat species become available, it will be useful to explore CHIRalphaEnv transcription in the context of broader physiological roles.”

3. L123-124: alpharetrovirus-like envelope ERVs; does this lineage stand for envelope or ERVs?

We realise that this naming could be misinterpreted regarding the retroviral classification as the alpha only applies to the envelope gene. We have therefore renamed the elements to CHIRalphaEnv to reflect that we are referring to only the envelope gene. The element has been renamed throughout the manuscript.

4. L201-203: I do not understand the logic behind this.

We thank the reviewer for highlighting the unclear nature of this section. As this is a relatively unique opportunity to have a collection of orthologous ERV elements in different species, we estimated a neutral rate of LTR mutation for these species. We have amended the text below to clarify:

“We used the LTR divergences and fixed common integration time of all CHIRalphaEnv7 elements to estimate the rate of neutral LTR mutation in Pteropodidae. With the given minimum integration time of 29.7 my between Cynopterus and Pteropus, we estimated an average mutation rate of 4.76855E-10 substitutions/site/year. Amongst pteropids, Cynopterus and Eidolon bats had the fastest mutation rates, whilst Pteropus and Rousettus had the slowest.

When applying the This estimated pteropodid rate of neutral LTR mutation is slower than the closest previously reported rate: the megabat neutral rate of evolution mutation (2.6x10-9).”

We have therefore simplified the text by emphasizing our main aim of providing a new rate estimation for LTRs in pteropodid bats.

## Reviewer 2

Re: Review of manuscript # PBiology-D-25-03002R1 for PLoS Biology "An ancient alpharetrovirus lineage in bats: evolutionary insights and possible roles in reproduction" by Emma F. Harding, Charles Reuben De Souza, Jose Gabriel Nino Barreat, and Aris Katzourakis.

Summary:

In this manuscript, Harding and colleagues present a paleovirology study in which they characterize an ancient endogenous retrovirus (ERV) lineage in the genomes of bats that possesses an env related to that of alpharetroviruses (alpharetrovirus-like env, or ARE). Building on an initial survey of six reference bat genomes (Jebb et al., 2020), the authors here demonstrate the presence of ARE sequences across 78 available bat genomes, including ~94 with ORFs that were associated with degraded gag and pol stretches, including ones that further group to one of several phylogenetically supported orthogroups by virtue of orthologous genome position. Analysis of the associated pol genes revealed similarity to non-alpha (spuma-like, gamma-like) ERV lineages that differed by orthogroup, suggesting dynamic evolutionary histories of the ancestral viruses contributing to the present ARE ERVs. Selection analysis suggests overall, a pattern of purifying selection, with specific sites pinpointed as having significant levels of positive selection. The authors demonstrate expression of the env from available transcriptome data, supporting expression of a spliced env transcript in endometrium of two pregnant Carollia bats; assembled transcripts aligned to an ARE ERV locus. Additional BLAST searches returned ARE-related sequences in saurian, amphibian, and mammalian genomes, some containing 'intact' ARE-related copies. Phylogenetic analysis places the bat ARE ERVs ingroup to saurian ARE-related ones, suggesting ancient cross-species transmission from saurian to chiropteran ancestors of the env.

**Comments:**

1. Five 'intact' AREs were identified in the initial search of bat genomes, from which one (P. Alecto) was then used to query available bat genomes. Why was that one (of the five) selected? Did that copy possess any distinguishing features that it was selected over the remaining four intact AREs?

We thank the reviewer for highlighting issues with the clarity and rationale of these methods. To address this, we redid the analysis using a sequence-agnostic approach: we searched all available bat genomes for any envelopes matching a custom trained alpharetrovirus HMM. Using this approach, we were able to reproduce our findings and find some additional sequences. This new method simplifies our approach and improves the clarity of the manuscript. We have amended the text in the methods section as follows:

“Retroviral proteins (n=193) spanning all extant and endogenous retroviral genera were used to query all chiropteran genomes (n=79) using a DIAMOND (61) search implemented in HI-FEVER.”

“A custom Hidden Markov Model (HMM) was trained on alpharetrovirus envelope sequences (n=6) using the HMMER tool ([http://hmmer.org](http://hmmer.org/)) and used to identify alpha-like envelopes in the HI-FEVER output.”

We have also amended the results section to reflect these changes:

“To identify Alpharetrovirus-like envelopes (AREs) in bats, we screened 79 available chiropteran genomes using the BLAST-based workflow HI-FEVER and validated hits with a custom Hidden Markov Model (HMM). AREs were detected in all 79 genomes comprising 2,740 total sequences of which 142 were intact and retained >300 aa ORFs, representing full-length envelope genes (Supplementary Table 1-3).”

We also redid Figure 2 to include the new sequences and have modified the results section describing this figure:

“Phylogenetically they form a sister clade to endogenous elements from lizards and caecilians (97% bootstrap support), which themselves are a sister clade to modern Alpharetrovirus sequences in birds (100% bootstrap support) (Figure 1). The Rhinolophus alpha-like sequence previously noted (19) clustered with these bat AREs, indicating they are from the same retroviral lineage (Figure 2). The 142 intact elements split into two clades: one comprised exclusively of Saccopteryx leptura and Saccopteryx bilineata sequences (n=39) that clusters with endogenous sequnces from lizards and caecilians. The other clade comprises the other 102 sequences spanning all chiropteran species surveyed and is the focus of further analysis.”

We have also created a schematic of the main methods that were used in this manuscript to improve clarity. This is now Figure 6 in the methods section.

2. Were any additional copies of the eight orthogroup insertions identified within the 1,463 total sequences that were not intact (at least by the criterion of at least 300 aa reading frames)?

We did search for degraded copies of the eight identified orthogroups through a BLASTn search of the elements and 40kB flanks (20kB each side) against the NCBI reference genome database. The results were difficult to interpret, likely due to the age of these elements and the variability and rearrangement of many chiropteran genomes, for example the high density of LINE and SINE elements in vespertillionidae. Any possible hits to orthogroups outside of the detected hosts from this study were cross-referenced with the degraded CHIRalphaEnv elements in supplementary table 10.

Overall, we found no evidence of CHIRalphaEnv orthogroups outside of the reported hosts from this manuscript. For example, CHIRalphaEnv 2 was not detected in any bat genomes aside from Vespertilionidae, Molossidae and Miniopteridae. Whilst this does not preclude them from existing, our methods used cannot detect any additional copies of the eight orthogroup insertions that were degraded.

3. If the goal of the search was to identify ARE env reading frames requiring >300 aa reading frame (essentially, env genes), it would help the reader if that was explicitly stated. For example, at line 136, adding "under criteria of…" and at line 151, "to expand the search", adding "for intact ARE envs" or similar.

We have simplified the results and rationale for more clarity (see comment 1). We now describe the 300 AA criteria explicitly in the results:

“AREs were detected in 73 genomes comprising 2,739 total sequences of which 142 were intact and retained >300 aa ORFs, representing full or near full-length envelope genes.”

4. In the initial results section, it might be confusing to introduce the Alpha-like envelopes (AREs), and then rename them CHIRalpha (for CHIRopteran alpharetrovirus). Why not refer to them as CHIRalpha [env] upfront? Along the same lines, it could be misleading to refer to the env genes alone as 'CHIRopteran retrovirus', when it is the gene (and not the ERVs themselves) that are alpha-like.

We agree that the previous naming could be consolidated for clarity. We retain the introduction of AREs followed by CHIRalphaEnv because we only designate a subset of the AREs identified as belonging to the CHIRalphaEnv lineage. This has been clarified in the manuscript below:

“The 142 intact elements split into two clades: one comprised exclusively of Saccopteryx leptura and Saccopteryx bilineata sequences (n=39) that clusters with endogenous sequnces from lizards and caecilians. The other clade comprises the other 102 sequences spanning all chiropteran species surveyed. We designated this clade CHIRalphaEnv (CHIRopteran alpharetrovirus Envelope) and focussed on it for further analysis.”

We have also renamed the CHIRalpha elements to be CHIRalphaEnv, reflecting that only the envelope gene is alpha-like.

5. Of the 94 intact ARE envs, eight orthogroups were identified. Maybe this was overlooked by the reviewer, but did any of the identified bats with an ARE env possess copies from more than one orthogroup? In other words, if a species identified has a CHIRalpha env, are the other env loci degraded (or absent)? For example, CHIRalpha3 and 5 in Phylostomidae bats.

We did observe bat species with multiple, intact CHIRalphaEnv elements from different orthogroups. To explicitly describe this, we have added the following text to the results:

“Interestingly, 25 bat species contained intact CHIRalphaEnv elements from multiple orthogroups (Figure 2 & Supplementary Table 1), indicating multiple co-option events within these genomes.”

6. Figure 3D. Which sequences are shown? Are these consensus ARE envs of each orthogroup or representative copies?

Figure 3D shows representative sequences from each orthogroup which can be found in Supplementary Table 2. The figure legend has been updated to reflect this:

*“D: Alignment of representative sequences from each CHIRalphaEnv orthogroup showing the fusion peptide and immunosuppressive domain (ISD).”*

We have also included our selection of representative sequences in the methods, stating that they were randomly selected within each orthogroup. The added text reads:

“Given that the elements of each orthogroup share the same canonical motifs and sequence structure, we randomly selected the representative sequences for use in further analyses, where applicable (Supplementary Table 4).”

7. At line 251: "All orthogroups… retained a 16-32 aa signal peptide…", is this meant that every ARE env possesses a signal peptide or a representative ARE env within that orthogroup? Does every copy of CHIRalpha1, 2, 3, 6, and 8 lack the furin cleavage site?

We thank the reviewer for identifying this and have clarified this in the manuscript. The signal peptide presence was based on a representative ERV from each orthogroup (listed in Supplementary Table 2). The results have been amended to clarify this:

“The representative of each orthogroup, with the exception of CHIRalpha2 in Vespertilionidae, retained a 16-32 aa signal peptide at the N terminus of the CDS (Figure 3, Panel D).”

For the furin cleavage site, we added its presence/absence to each CHIRalpha sequence in Fig 2, denoted by a red circle next to the sequence label.

8. For the sites found to be under positive selection, is there any indication of how they might impact the folding or functions of the product?

We thank the reviewer for this question and agree that it is an interesting question to consider. We closely examined the physiochemical properties of these sites and added the following text to the results section:

“At these four sites we observe no preference for hydrophobic/hydrophilic or polar/non-polar residues, indicating a relaxation of structural and/or functional restraints on these residues.”

We also generated a structure of the CHIRalphaEnv protein using Google ColabFold servers (based on Alphafold 2), however the pLDDT confidence scores were too low for us to make any conclusions on the impact of altering these sites on the structure and folding of the protein. This has not been included in the manuscript due to the low confidence. We look forward to exploring this more as modelling improves and more structures become available.

Currently, as alpharetrovirus envelope mechanisms and functional motifs have not been well characterised and their behavior/interactions in mammalian cells are unclear, we are hesitant to speculate on the implications of these sites without experimental data or observations.

9. The reviewer is not a bat expert, but a quick search reveals Carollia belong to Phylostomidae. Is the reconstituted ARE env transcript of the CHIRalpha 3 or 5 (and which ARE env of the two >300 aa does it map to in Table S1)?

We thank the reviewer for highlighting this ambiguity and have addressed it by adding the following text to the results:

“Of the two intact CHIRalphaEnv elements in the Carollia perpicillata genome, the endometrium transcripts originated from the orthogroup 5 element (PVKM010017682.1:9546-10649). No evidence of transcription from the orthogroup 3 element was observed.”

10. Along the lines of these questions/comments as well as above, a table in the main text may be of help for some clarification for the audience pertaining to the ARE envs, orthogroups, and orthogroup properties.

We thank the reviewer for their suggestion and agree that a single place summarising orthogroup information is beneficial. To address this, we have made Figure 2 a reference for all orthogroup information. On top of the existing tree, we have added the presence/absence of the RXKR motif and LTRs to allow for easier cross-referencing between the manuscript, figures and tables.

11. The text near line 253 indicates "Not all orthogroups retained the RXXR furin cleavage site…" among ones lacking the furin cleavage site are CHIRalpha3. Does the expressed reconstituted transcript map to an ARE env with a predicted furin cleavage site?

The transcribed orthogroup 5 element does not have a predicted furin cleavage site, indicating it is unlikely to function as a canonical retroviral envelope. To better clarify this, we have overlaid the presence of furin cleavage sites on Fig 2.

12. Line 479: "infecting". Suggest "present in".

We have followed the reviewers’ suggestion and change the text to the following:

“Although no extant bat retroviruses with alpharetrovirus envelopes have been isolated, it would be useful to determine if this lineage is still present in bats or has since become extinct.”

13. Line 483: "it would be interesting to determine if…". Suggest adding a statement here to reflect Jebb et al., in which they state (referring to ERV sequences) "Phylogenetic analysis suggests that most viral integrations are relatively recent integration events ", noting that they performed a pol-based analysis (the reviewer does not know -did they include any ERVs with the ARE envs?).

We thank the reviewer for this suggestion and have added some discussion regarding the age of these elements in relation to other bat ERVs. The text now reads:

“Most characterised bat ERVs are relatively young, with ERV integrations concentrated between 10-40 million years ago (19, 38, 42, 43). With the exception of orthogroup 6 and 7, CHIRalphaEnv elements are predicted to be older than 40 million years (95% confidence interval) and represent some of the oldest cross-family orthologous ERVs identified in bats.”

We agree that it is a very interesting point that most viral integrations in bats are recent. As many papers do purely pol-based analyses, it is difficult to determine if they have detected anything with CHIRalphaEnv envelopes. To our knowledge only two papers report AREs in bats (Jebb et al. and <https://www.sciencedirect.com/science/article/pii/S0168170224001771>), neither of which specifically comment on their estimated age or integration times. Additionally, more recent ERVs can be unfixed and variable within a population, not necessarily represented in the reference genome. This is definitely an interesting avenue to pursue and we hope to continue exploring these CHIRalphaEnv elements in the context of all bat ERVs in the future.

14. Lines 409-410: "with evidence of co-option at least eight times". Suggest removing, as this evidence is brought up later in the same paragraph.

We have removed the suggested text. The sentence now reads:

“We describe an unrecognised ancient lineage of alpharetroviruses that have persisted within Chiroptera for more than 45 million years.”

15. Lines 417-418: "…to perform a vital host function". For clarification, was each of the ARE env orthogroup ancestor integrations fixed for benefit of the same vital host function? Are all ARE envs of the same orthogroup proposed to perform the same vital host function?

We speculate that each CHIRalphaEnv sequence performs the same host function, with the exception of CHIRalphaEnv1 based on selection analysis results. The remarkable conservation of at least one CHIRalphaEnv in each bat genome and their similar selection patterns suggest they perform the same function. However, the presence of intact elements from multiple orthogroups in some bat species suggests either redundancy of function or that one orthogroup is in the process of being replaced by another. Until more samples or transcriptomes become available it is difficult to speculate on the exact function and whether they are all co-opted for the same reason. We look forward to future studies exploring this as more data becomes available.

16. Lines 425-426: "highly active genomic regions" vs "much more stable regions". Is there evidence of the genomic regions being "highly active" and what is meant by this term? Is this to refer to an abundance of TEs (and does that property lend that region to be highly "active"?)? Likewise, are the other regions much more stable due to the lack of observed TEs?

In this context, we do refer to regions as genomically active based on the number of TEs in the area and therefore the historical turbulence of this region. Stable regions, as observed for the pteropus CHIRalpha elements, provide much easier orthology and LTR identification due to the lack of integrations and rearrangements surrounding the ERV. In contrast, the “active” regions have had much TE activity to rearrange and interrupt the flanking regions, making genomic analysis much more difficult for these ERVs.

To clarify our use of these terms in the manuscript, we have amended the relevant sentence to now read:

“…were integrated into highly active genomic regions (containing many transposons), whilst others like CHIRalphaEnv7 seemed to be in much more stable regions (few to no transposons).”

17. Line 431: Suggest breaking this sentence into the next paragraph.

We have followed the reviewer’s suggestion and changed this into two paragraphs.

18. Lines 465-467: "The best characterized syncytins… are derived from gammaretroviral envelopes, specifically ones similar to type-D betaretrovirus envelopes (Fig. 1)" -as meant?

We thank the reviewer for highlighting this confusion – the confusion arises from some betaretroviruses containing gamma-type envelopes (GTEs) that phylogenetically cluster with gammaretroviruses. We have clarified this in the text by referring to the envelopes as gamma-type rather than gammaretroviral and by specifying that Type-D betaretroviruses are recombinants. The text now reads:

“The best characterised syncytins, syncytin-1 and -2 in humans and syncytin-A and -B in mice are derived from gamma-type envelopes, specifically ones similar to type-D recombinant betaretrovirus envelopes (Figure 1).”

19. Line 488: "Most…". If the 7/8 gamma(ARTgagpol)-like/alpha-like are recombinant, are the remaining orthologue ERV-L(spuma)-like/alpha-like not as well?

We thank the reviewer for highlighting this discrepancy – originally our statement the “most” encompassed the related sequences in saurians as well which was unclear. We have amended this statement to improve its clarity, it now reads:

“All CHIRalphaEnv orthogroups were recombinant retroviruses, with env sequences of alpharetroviral origin and gag/pol regions more closely related to the gammaretroviruses (n=7) or ERV-L retroviruses (n=1).”

## Reviewer 3

In their article entitled "An ancient alpharetrovirus lineage in bats: evolutionary insights and possible roles in reproduction" Harding et al… detail an ancient alpharetrovirus lineage within bats, that they argue was transmitted from reptiles to mammals and ubiquitously infected bats for over 23 million years.

These results are based on comparative genomic screening of 820 vertebrate genomes, 60 bats and 742 other vertebrate genomes, where the researchers used their custom EVE discovery Nextflow workflow "HI-FEVER" to identify alpha-like envelope genes integrated into the vertebrate genomes. They next identified if these insertions were orthologous across taxa, assessing the flanking regions around the insertion sites to identify if the ERVs shared the same proximal up- and down- stream annotations and also generating orthogroups using a method they published in Nature Microbiology 2024. They extracted 10kb and 20kb flanking regions around the envelope insertion site and searched for gag/pol retroviral core genes and long terminal repeats and used these hits to identify the retrovirus type they originated from. They searched for signal peptides, transmembrane domains, furin cleavage sites, fusion peptides and immunosuppressive domains (ISD) in the envelope coding regions
 extracted. They aligned orthogroups and ran selection test across these groups to estimate evolutionary selection pressures acting on these inserted elements. They further explored if the bat alpharetrovirus envelope sequences (CHIRalpha) were expressed by searching the SRA repository. They aligned all (?) elements recovered and generated phylogenetic trees using ML and inferred timetress using BEAST and also estimated bat divergence times based on Timetree.

Overall these are really exciting results. These findings challenge current prevailing hypotheses regarding the host specificity of alpharetroviruses, they suggest multiple cross-class transmission events from reptiles into mammals. They suggest a more ubiquitous exaptation of these retroviruses in mammals and suggest their role as synctins in bat reproductive biology. These results are only now evident from the wealth of new vertebrate genomes published through large sequencing consortia, VGP, Bat1K, Zoonomia, DToL etc. and the pipelines developed by this team. These results highlight the new insights we can now find with our large scale genomic resources.

However as written, it is very difficult to judge the validations of these claims. The methods are lacking much detail regarding the data sets, analyses, methodologies used. The results are not clear as presented. The figures are not labelled in enough detail and do not contain all of the information needed to properly assess these results. Some bold claims that are made need further exploration as they could be interpreted in a different manner.

Below I list the places where I found things difficult to follow or question some of the claims. These suggestions are made to improve this paper and make it more accessible to the reader and to present better, the necessary results to back up these exciting claims.


Introduction:

1. Line 82 "Alpharetrovirus ERVs are particularly scarce and are the only genus not yet identified in mammalian genomes (14-16)."

   This is not the case, they were identified in bat genomes in 2020. (Jebb et al Nature 2020). Perhaps the authors mean full length RVs but this should be clarified here and is a little ambiguous and a little misleading.

We thank the reviewer for highlighting the possibility of misinterpretation and have amended the manuscript to improve clarity. We have added the following sentences to clarify that we define classification based on the polymerase gene, and the complexity that arises from recombination with different envelopes:

“Classification of a retrovirus is based primarily on the polymerase gene, as retroviruses frequently recombine to acquire new envelopes which can complicate classification.”

“Retroviral envelopes often have distinct evolutionary histories to polymerases, recombining indiscriminately to confer new host ranges and physiochemical properties to a retroviral lineage.”

We have also modified the text in the introduction to better describe the previous findings in Jebb et al. and Lamba et al. and highlight the research questions that arise from their studies.

“AREs have been detected in eleven bat families spanning much of the known bat diversity, indicating they were prevalent viral pathogens within the order Chiroptera (20). These findings pose many new questions: namely are these bat AREs true members of the Alpharetrovirus genus that once circulated more widely or are these AREs simply the closest endogenous relatives.”

2. Line 103 Define "syncytiotrophoblast" it will make it easier for readers to follow.

We have added a definition as suggested by the reviewer. The text now reads:
“contributing to formation of the syncytiotrophoblast – the multinucleated, epithelial layer of placental villi”

Results:

3. 134" We initially screened Yinpterochiroptera bat genomes for Alpharetrovirus-like envelopes (AREs) where they had been previously reported."

   This statement is misleading. Only the family Pteropodidae was screened initially for AREs? My understanding from the methods was 18 pteropodids were studied. So I am a little confused as why the authors would have done this? There were other yinpterochiropteran genomes available at the time (July 2024). The first ARE was found in Rhinolophus bats? Why not include the Rhinolophoidea or other non-pteropodid genomes. This needs to be clarified and explored. Pteropodidae are one of the more derived families of bats why pick them for the initial screening?

We agree that the rationale and methodology was unclear and have simplified the methods to a single HI-FEVER search through which we reproduced and expanded our initial results. Our strategy now involves a comprehensive initial screening across all available bat genomes. Please see Reviewer 2 comment 1 for more detail.

4. It is this lack of detail that makes it hard to follow the rationale for some of the decisions taken. Why those 18 bat genomes? Why not others? The rationale for species inclusion/exclusion in datasets analysed throughout needs to be detailed.

We agree and have modified the methods in favour of a more comprehensive, robust and reproducible search strategy. Please see Reviewer 2 comment 1 for more detail.

5. 153 "AREs were detected in all 78 chiropteran genomes comprising 1,463 total sequences of which 94 were intact and retained >300 aa ORFs (Supplementary Table 1 & 3). These elements were called CHIRalpha
    (CHIRopteran alpharetrovirus)".

    - Need a link to these datasets so they can be downloaded and explored.

We have uploaded all sequences and metadata to the Open Science Framework repository (https://osf.io/jc2g4/overview?view_only=4d4c4d99f2984a1f884080f5cb3efc17) so they are publicly available.

6. 160 "We identified eight main orthogroups (CHIRalpha 1-8) encompassing 89 of the 94 intact CHIRalpha elements >300 aa (Figure2)."

    -Need link for the orthogroups datasets also for the same reasons as above. There needs to be more detail of the methods used to generate these orthogroups and more descriptions of the orthogroups themselves. There should be a table identifying which genome and bat a particular alpha element came from.

We have uploaded the orthogroups datasets to the Open Science Framework repository. We have also amended Supplementary Table 3 to convey the intact elements and associated data (eg. Which species they were found in and which orthogroup they belong to).

7. Also from Figure 2 and the results section, I am not sure which bat species belongs to which orthogroup/CHIRalpha element group. For example CHIRalpha5 is argued to be only detected in Phyllostomidae bats, however it also appears to be found in Noctilionidae and Mormoopidae? The Molossidae clade is grouping with Miniopteridae (98%),which is an interesting grouping. The Miniopteridae are sister to Vespertilionidae. Is Miniopteridae included in the CHIRalpha4 group or in the CHIRalpha 2 group? CHIRalpha 1 is not found in all Rhinolophus bats, it is missing from Rhinolphus-alpha-like species (???) listed in the CHIRalpha8 group. What is this branch, which species etc.??, this tip label is different to everything else.

We thank the reviewer for highlighting this confusion and have clarified the text, figure and supplementary table. In Supplementary Table 1 we have added the orthogroups detected in each bat genome as a new column.

The rhinolophus alpha-like sequence was the detected envelope from the Jebb et al. paper and was included to demonstrate that it is a CHIRalphaEnv element. To avoid confusion, we have removed this sequence from Figure 1 and better described its relationship to CHIRalphaEnv in the text. The text now reads:

“The Rhinolophus alpha-like sequence previously noted (19) clustered with these bat AREs, indicating they are from the same retroviral lineage (Figure 2).”

8. The authors need to identify which branch on the tree they are naming as a CHIRalpha group. This would be easier to understand rather than highlights around tips, then refer to this in the text and in a table as mentioned above.

We thank the reviewer for this suggestion and have moved the CHIRalphaEnv labelling from the tips to the branches in Figure 2. We have also clarified which elements are defined as CHIRalphaEnv (see Reviewer 2, comment 4).

9. Also they need to double check their bat phylogeny and family level representatives included in these families, especially as they will be using bat time trees to estimate dates of insertion and orthology. Looking at figure 2, I would question if the authors consider Pteronotus as a phyllostomid and Miniopterus as a molossid. This needs to be clarified and the naming and inclusion of bat species in these orthogroup needs to be better defined.

We thank the reviewer for highlighting this challenge with bat taxonomy. We addressed this by using the NCBI taxonomy database as a guide for the current classification of each bat species in this study and have updated both Figure 2 and Supplementary Table 1 to reflect this.

10. I would like to see some evaluation of the quality of the genomes used. Many were Ilumina short read genome "scans" others were long-read based reference genomes. Was there a difference in ERV recovery? Could this bias the results?

We thank the reviewer for raising this concern and agree that genome quality can greatly influence ERV recovery and conclusions. We have added genome assembly completeness to Supplementary Table 1. Assembly quality and completeness can affect the overall count of CHIRalphaEnv elements (intact and degraded), and we expect these absolute numbers to change between genome versions. However, as the focus of our study is on comparing the intact elements, we do not believe that genome quality will affect the main results and conclusions of this study. We identified at least one intact CHIRalphaEnv element in 73/80 genomes and were able to assign 89 of the intact elements to orthogroups based on flanking DNA, providing enough sequence and orthology information to support our conclusions.

11. Also the annotation across vertebrates genomes is also at different levels of resolution? How did the authors account for this when looking at syntenic insertions across vertebrates? Even within the bat genomes alone there are different annotations, which change depending on the annotation method used, and there can be problems with gene names? How did the authors account for this?

To account for different levels of genome annotation, we used two methods to identify and confirm orthology. The first method was annotation-independent, relying on BLAST comparisons between flanking 30kB nucleotide regions. The second method was manual and involved identifying which genes were closest upstream and downstream to the integration site. By combining both methods we were able to confirm orthology of each element regardless of annotation or genome quality.

We have clarified this by adding more detail in the methods section regarding the manual identification of orthology in less-annotated genomes.

“As a second, independent test of orthology, proximal host genes upstream and downstream of the integrated ERVs were identified using NCBI genome data viewer on well-annotated bat genomes. The protein sequences of these proximal genes were used for tBLASTn searches to other, less annotated, bat genomes and cross-referenced with the CHIRalphaEnv loci. Relative positions of host gene matches and absolute distance from the insertions was used to confirm orthology between bat genomes.”

12. 176 "Yanchiroptera CHIRalpha elements grouped into five orthogroups".

     -The subordinal group is Yangochiroptera not Yanchiroptera, this needs to be corrected throughout.

We thank the reviewer for noting this and have corrected the text as suggested.

13. 196- "We estimated the minimum integration time of each orthogroup based on their host divergence dates and LTR divergence dating where possible. CHIRalpha1, 2, 3, 4, 5 and 8 were the oldest, all integrating between 36.46 and 43.80 million years ago (Supplementary Table 2 & Supplementary Figure 2)"

     -The authors need to clarify where the dates for the divergence of their bat phylogeny come from (ie Table S2 column I). Rhinolophidae for example, were the same bat species included in the timetree.org estimation as the authors have included in this study? This is important as it has direct implications for their time estimations for CHIRalpha circulation. Is the bat tree used in Timetree the most up-to date? These methods need to be critiqued and better explored, ultimately providing a range of dates. The time tree should be included in the supplemental. However, probably the best dated tree and bat phylogeny, which include most of the taxa they have studied is Foley et al Science 2023, from the Zoonomia consortium. I would recommend they used these dates and phylogeny throughout.

We thank the reviewer for this comment and have clarified which dates were used and how they were sourced.

We used timetree.org to generate a timetree of the bat species used in this study. The divergence time of chiroptera is similar between Timetree and Foley et al (~62-63MY) directly succeeding the KPg boundary. Additionally, for the species divergences analysed in Foley et al, the Timetree estimations all fall within the confidence interval for these times. We have added this in the text for methods validation, as well as comparing it to other estimates of mammalian divergence times. The methods now read:

“Bat divergence times were derived from timetree.org (85) which compiled information from multiple studies to provide median divergence times and confidence intervals which were used for prior calibrations of the BEAST2 timetree. The divergence times and confidence intervals compiled by timetree are consistent with previous estimates of mammalian evolution. The tree used and estimated divergence times are available as Supplementary Figure 9 and in Supplementary Table 4 respectively.”

14. For dating the ERV insertion times the authors used timetree.org calibrations of bat divergence. I would like to see a table listing these divergence splits and the phylogenetic bat tree that was used? If the tree is wrong or the estimates are incorrect this will directly affect the interpretation. As written it is not easy to follow and I can't assess if these splits are based on the most up to date estimates and trees.

We thank the reviewer for raising this important point. We agree that incorrect dates and phylogenies can affect the interpretation of CHIRalphaEnv evolution and have provided the timetree that was used for all calibration times and conclusions as a Supplementary figure to aid reproducibility and transparency. See previous comment for changes to the methods text. We have also provided the studies used to generate the timetree.org estimate for each node that was used as a calibration date in our study. For example, the estimate of 29.7MY divergence time for Pteropodidae was derived from three studies spanning 1997-2014 (Fritz et al. 2009, Hollar et al. 1997 & Yu et al. 2014) which have now been referenced in Supplementary Table 2. Links to these papers are provided in Supplementary Table 4 next to their estimated dates.

These age distributions are taken into account in the priors for the Bayesian inference in BEAST and should minimize discrepancies between timetree estimations and those of individual studies used in the compilation of the timetree (including Foley et al.). The divergence times from TimeTree were used to provide a log-normal distribution of estimated integration time as a prior which BEAST could then resample throughout the analysis. The final dates provided are always estimates and we acknowledge that they may change as more information becomes available in the future.

15. 227 "When applying the megabat neutral rate of evolution (2.6x10-9) (36) to CHIRalpha7 we estimated integration dates between 6.5-18.3 million years ago which is inconsistent with the divergence time of the hosts (~29.7 million years)(37).

     -Why use this estimate of evolution rate? All of these estimates are based on old data sets and papers published nearly >20-25 years ago? Newer phylogenomic papers and divergence date estimates should be used e.g. Foley et al Science 2023.

We thank the reviewer for raising this point and the issues with evolution rate selection. As we specifically need the divergence time for the genera within orthogroup 7 (*Pteropus, Cynopterus, Rousettus* etc), that limited the studies that we can draw from. For example, Foley et al. does not provide an estimated time for divergence within the pteropodid group (eg. *Pteropus* vs. *Rousettus* vs. *Cynopterus*) and therefore cannot be used to predict a minimum integration time using our methods.

To our knowledge, not many studies have been conducted on the evolutionary rate of bats specifically, especially megabats. The megabat neutral rate of evolution (Cantrell, 2008: Loss of LINE-1 Activity in the Megabats) was selected as it was calculated specifically on the hosts of orthogroup 7 and was therefore the most relevant. As an alternative we also derived our own rate estimation based on the LTR divergence of each element, see Reviewer 1 comment 4 for more detail.

16. 296 "… 206/208) and two of which are in the cytoplasmic tail beyond the transmembrane domain
     (sites 378/389) (Supplementary Table 6)"
     - Should be supplementary table 7 not 6?

We have changed the text as suggested – it now reads:

“...and two of which are in the cytoplasmic tail beyond the transmembrane domain (sites 378/389) (Supplementary Table 7).”

17. 313 " To search for transcription and therefore possible host function, we screened 1,982 bat RNA-Seq experiments from a range of bat species (n=110) and tissues (35 categories) (Supplementary Table 5)."
     -Should be supplementary table 6 not 5?

We have changed the text as suggested – it now reads:

“...experiments from a range of bat species (n=110) and tissues (35 categories) (Supplementary Table 8).”

Transcriptome analyses-

18. What I find strange in these results is that the endometrium and the foot show high expression (even though only based on 2 samples). Why then did the authors consider only its role in reproduction? How can its expression in the foot be accounted for?

We thank the reviewer for raising this point. We primarily considered the role of CHIRalphaEnv in reproduction and development based on its (relatively) specific expression matching that of previously described developmental ENV co-options in literature. Both of the foot samples were from a bat infected with white-nose syndrome. Unfortunately, as no “healthy” foot samples were available for screening, it is impossible to tell if CHIRalphaEnv transcription is normal in bat feet or a result of dysregulation from the fungal infection.

We have added the following text to the discussion regarding this point:

“Envelope co-option has been utilised for a myriad of functions within vertebrates, most notably for antiviral defence or development of the placenta. The observed prevalence of CHIRalphaEnv transcripts within endometrium samples and low expression in all other tissues would suggest the element has a role in placentation or development. However, this hypothesis cannot explain the heightened transcription detected in foot samples from a *Myotis myotis* bat. This bat was infected with the fungus Pseudogymnoascus destructans which causes white-nose syndrome (WNS), a potent pathogen in hibernating bats. *Myotis myotis* show little transcriptional dysregulation during WNS infection (49), however it is possible that CHIRalphaEnv is activated as an immunomodulatory protein in response. However, at least 51 of the RNA-Seq experiments were from bats with WNS and only 12 contained detectable levels of CHIRalphaEnv expression (Supplementary Table 8), indicating that CHIRalphaEnv expression is not reliably correlated with this disease.

Similarly, a further 47 RNA-Seq experiments were from bats infected with various viruses, of which only six displayed CHIRalphaEnv expression. The lack of expression in response to infection and absence in immune tissues (eg. Spleen, lymph nodes) precludes the antiviral function of CHIRalphaEnv based on current knowledge (50, 51). We therefore focus on the possible developmental role of CHIRalphaEnv elements based on endometrial expression.”

.

19. Also I think it is a leap to conclude that these elements are indeed syncytins. This needs to be better explored across bats with much wider focus on placenta like transcriptomes. Their conclusions need to reflect this or they needs to discuss the further step required to validate their hypotheses.

We thank the reviewer for raising this point and agree that syncytins are one, but not the only, possibility for CHIRalphaEnv function. We have added discussion around alternate functions (see reviewer 3, comment 19) and also addressed the limitations of making hypotheses from limited sample sizes. We have added the following text to the discussion:

“Due to the limited sample sizes for some tissues (eg. Only two samples for both foot and endometrium), unambiguously suggesting a function to CHIRalphaEnv elements is challenging. As more RNA sequencing and proteomes from different tissues and bat species become available, it will be useful to explore CHIRalphaEnv transcription in the context of broader physiological roles.”

We also added experimental work inducing CHIRalphaEnv expression in bat cells (see Reviewer 1, comment 2) to explore their function as fusogenic syncytins. We also find the parallel between menstruation and intact CHIRalphaEnv elements intriguing and highlight that as a major discussion point.
We agree that continued exploration of this as more samples become available is vital to better understand the function of CHIRalphaEnv elements. We have added the following to the discussion to comment on the future directions required to continue exploring this hypothesis:

“Further exploration of CHIRalphaEnv expression as bat transcriptomes and proteomes become available, particularly from reproductive organs, is vital to understanding the function of these elements across Chiropterans.”

20. 329- "Expression levels were the highest in the two endometrium samples from pregnant Carollia bats with 0.007% and 0.009% respectively of total reads mapping to CHIRalpha (Supplementary Table 6)."

     -Overall there is little expression in the SRA databases. Is this to be expected?

It is difficult to predict what is expected, however as the CHIRalphaEnv elements are ancient integrations and retain properties of retroviral envelopes we would not expect high expression as they may modulate the immune response, either through immunogenicity or immunosuppression. In general, ERV envelopes tend to be rapidly degraded in the genome due to detrimental effects of their expression. Based on this, low expression levels and expression only in select tissues is not unexpected.

21. 356 " determine the host range of CHIRalpha elements, we performed a DIAMOND
     BLASTx search using the CHIRalpha7 representative sequence to query available NCBI RefSeq vertebrate genomes (n=742)."
     - Please clarify why CHIRalpha 7 was used as a query?

We thank the reviewer for noting this. For this search, CHIRalphaEnv 7 was selected as it was comparatively well-preserved within the genome compared to other CHIRalphaEnv orthogroups. The BLAST search is robust to query selection as the CHIRalphaEnv elements are so similar between orthogroups – we obtained the same results when using different CHIRalphaEnv queries (with slightly different percentage identities).

22. Figure 5. Please indicate the length of the alignment that was used to make these trees. Also please provide the alignments files to enable more detailed investigation. The phylogenetic conclusions are based on homology of the alignments and can easily be misled. Therefore it is important that a reader can assess the alignment quality.

The sequences, alignment and tree used to produce Figure 5 have been uploaded into the Open Science Framework repository associated with this manuscript.

23. Their conclusions regarding the phylogenetic placement of the CHIRalpha lineage within a clade containing saurian, crocodilian and amphibian retroviruses depends on their confidence of their phylogeny. For these reasons we need to have confidence in the alignment, the homology and the support, therefore the alignments need to be available.

We thank the reviewer for this concern and have uploaded the sequences, alignments and tree files to the Open Science Framework repository associated with this study.

24. Also I suggest that they should run independent phylogenetic test, such as KH-SH, AU tests to explore the phylogenetic support for alternate topologies to strengthen their conclusions. This would give further support for their exciting claims.

We thank the reviewer for this suggestion and have performed topology tests to better support our conclusions. We used both BEAST and iqTree to perform testing and have included the alignments, constraints and resulting trees in the OSF repository.

We conducted AU, KH and SH tests in iqTree2 comparing the likelihood of a null hypothesis where sequences could be explained by host co-divergence to the unconstrained tree placing CHIRalphaEnv within the Alpharetrovirus genus. These tests rejected the null hypothesis, providing evidence of at least one cross-kingdom transmission between mammals and reptiles within alpharetroviruses.

We also constructed Bayesian analysis using BEAST with these sequences to provide an alternative estimation method for the root of the tree and the topology. The resulting BEAST tree demonstrated the same topology as the unconstrained maximum likelihood tree from iqTree, with mammalian CHIRalphaEnv sequences branching from within a saurian clade.

The methods have been described as below:

“Topology testing of evolutionary hypotheses was conducted in iqTree2. A constrained tree was generated placing the mammalian alpharetrovirus-like sequences as a monophyletic sister group to alpharetroviruses, with gammaretroviruses as an outgroup. The log-likelihood of this tree was compared with that of an unconstrained tree using iqTree2 with the RELL approximation and 10,000 replicates.

In BEAST, the same alignment was used to construct a Bayesian tree using model JTT+G4 with estimated invariable site proportions and gamma shape and an optimized relaxed clock and a Yule model.”

These results are summarised in the text below:

“To explore the evolutionary scenarios that explain this host distribution, we constructed constrained trees in iqTree2 and undertook maximum likelihood topology testing. The null hypothesis, where alpharetrovirus diversity is explained by host codivergence between mammals and reptiles, was rejected with p-values of 0.0005 in the KH and SH tests and 0.000809 in the AU test. The favoured hypothesis was a topology placing mammalian alpharetrovirus sequences nested within the reptilian sequences, indicating at least one cross-class transmission between reptiles and mammals has occurred to explain these observations.”

25. Figure 1, Please include the names of the orthogroups/lineages in the CHIRalpha clade. It is very odd to have no labels on the tips? There six branches but 8 CHIRalpha groups (?) so it is confusing as to what is being depicted? Also this figure seems to be a mixture of lots of orthogroups, single elements etc., previously published data, newly mined data for this study, so it is just not clear what has been used to make this tree. This needs further explanation in the methods, and also the alignment file used to generate this tree needs to be made available.

Originally figure 1 was created to show the relationship between CHIRalphaEnv sequences and other retroviral envelopes and we only used a subset of CHIRalphaEnv to demonstrate this. However, to avoid confusion, we have remade the figure to include all CHIRalphaEnv orthogroups. We have also changed the tree from a rooted (with the alpha/gamma split as the root) to an unrooted tree to better convey the relationship between CHIRalphaEnv elements and other retroviruses and ERVs.

We have uploaded the alignment and sequences used in this tree to the Open Science Framework repository associated with this paper.

The figure legend has been modified to reflect the multiple origins of sequences used within this tree and now reads:

“*Figure 1: Phylogenetic relationship of CHIRalphaEnv elements to other retroviral envelopes and ERV element*s. Amino acid sequences of circulating retroviruses, envelope-derived endogenous elements and syncytins were compiled from NCBI databases and literature.”

26. Sup Figure 1, there seems to be missing CHIR Orthogroups. Only 6 are listed here? Should there not be 7? (see line 189), 3 and 4 are not present?

Supplementary Figure 1 was based on polymerase gene fragments to reconstruct phylogeny. For both orthogroups 3 & 4 we could not detect a long ORF with enough overlap with the other sequences to be phylogenetically informative. We have clarified this in the text, which is modified below:

“we constructed phylogenetic trees of the reverse transcriptase (RT) domain of each pol (where available, 6/8 orthogroups).”

27. The methods and overall paper would benefit from a figure describing the pipeline of what their methods and what have done, which datasets were used to generate trees, which phylogenomic methods were used, which taxa were included etc. There are many, many different data sets, bats, mammals, ERVs, LTRs, envelopes etc. a simple schematic would make this easier to follow for everyone including the software they used for analyses.

We thank the reviewer for this suggestion and have created a methods schematic that outlines the core approaches and software used throughout the manuscript. This is now in the methods section as Figure 6.

We have also generally amended the methods to improve clarity (see Reviewer 2 comment 1) and uploaded all datasets to the associated OSF repository.

28. The datasets and alignments needs be placed in a public repository that is accessible for download and analyses.

We have created an Open Science Framework repository into which we have deposited all data associated with this paper, including alignments, sequences and raw data tables.

29. I would like to see a little more discussion of the point they raised in the introduction, line 114, "that retroviral envelopes have also been co-opted for anti-viral defence against exogenous viral infection." Perhaps this role could better explain the expression signature or overall prevalence of CHIRalphas in bats?

We thank the reviewer for this suggestion and have further considered CHIRalphaEnv as a potential antiviral-defence molecule. We previously had omitted discussion on this point, but have now added it to the text to comprehensively explain the possible functions of CHIRalphaEnv based on our current knowledge. See reviewer 3, comment 18 for the new discussion paragraph addressing this.
