# Supplementary material for: An ancient alpharetrovirus lineage in bats: Evolutionary insights and possible roles in reproduction
Source: PLoS Genet. 2026 Aug 25;22(8):e1012277. doi: 10.1371/journal.pgen.1012277 (PMC13568524; doi:10.1371/journal.pgen.1012277)
Supplement: Peer review history

# Author comment: Author response to Decision Letter 0

**DOI:** 10.1371/journal.pgen.1012277.r001

**Transfer Alert**

This paper was transferred from another journal. As a result, its full editorial history (including decision letters, peer reviews and author responses) may not be present.

15 Jun 2026

Attachment Submitted filename: Response to Reviewers-4.docx

# Editor report: Decision Letter 0

**DOI:** 10.1371/journal.pgen.1012277.r002

**Authors:** Justin Fay, Harmit Malik

23 Jun 2026

PGENETICS-D-26-00624

An ancient alpharetrovirus lineage in bats: evolutionary insights and possible roles in reproduction

PLOS Genetics

Dear Dr. Katzourakis,

Thank you for transferring your manuscript to PLOS Genetics together with the reviews from its previous evaluation. I have now carefully read the manuscript, the multiple rounds of three reviews, and your detailed point-by-point responses.

I am pleased to say that the work is, in my assessment, well-suited to PLOS Genetics and is close to acceptable. The cross-class transmission analysis, the identification of eight independent co-option events, the divergence dating, and the new fusogenicity assays collectively move the work substantially beyond the initial observation of alpha-like envelopes in a handful of reference genomes (Jebb et al. 2020) and address the reviewers' principal scientific concerns. I am therefore not requesting any new experiments.

I am returning the manuscript for a round of **minor revision** . The required changes are matters of internal consistency and interpretive framing rather than new data, but several of them are essential and should be addressed in full before the paper can be accepted. They are itemized below in order of priority.

**1. Reconcile the syncytin framing with your own negative fusogenicity result.**  This is the most important point. Your new experiments found no syncytia across the four cell lines tested, and you now correctly describe the elements as "non-fusogenic" in the Results and in your cover letter. However, the manuscript continues to advance canonical syncytin language elsewhere: the Abstract proposes "the first mammalian syncytin candidate," the Discussion (around line 503) states that "CHIRalphaEnv are the first mammalian syncytin candidates related to alpharetroviruses," and "syncytin" remains a listed keyword. A non-fusogenic element does not meet the standard functional definition of a syncytin, so as written, these statements are internally inconsistent with the data.

Please resolve this throughout the manuscript by either (a) replacing "syncytin candidate" with more accurate language, such as "co-opted/exapted envelope with a likely reproductive role," or (b) if you wish to retain a syncytin-related framing, explicitly defining a non-fusogenic, syncytin-*like*  category (**note that I do not favor this unless you can appropriately justify this; the two mouse syncytin examples you cite are less clear about this dichotomy** ) and justifying it against the conventional fusogenicity criterion, with the negative assay foregrounded rather than downstream. The hedged title ("possible roles in reproduction") is appropriate and may stand; the Abstract and Discussion need to be brought into line with it.

**2. Make the genome and element counts consistent across the entire manuscript.**  The methodological overhaul has left the numbers unreconciled. The number of genomes screened appears variously as 78, 79, and 80 (e.g., "n=79" in the revised Methods versus "80 available chiropteran genomes" at Results line 112). The number of genomes with detections appears as both "all 79 genomes" and "73 genomes" (Results line 115). The count of intact >300-aa elements appears as 141 (Results line 115), 142 (elsewhere in the Results and in your responses), and 94 (legacy figure). Please choose the correct, current values and apply them uniformly across the Abstract, Results, Methods, all figures, and the supplementary tables, and confirm that "detected in all genomes" versus "detected in 73 genomes" is stated consistently.

**3. Verify every figure and supplementary table cross-reference.**  Several supplementary tables were renumbered during revision (e.g., the reviewer-flagged corrections of Table 5→8 and Table 6→7). Some in-text citations still appear to point to pre-renumbering targets. Please perform a complete pass confirming that every Figure and Supplementary Table/Figure callout in the text resolves to the intended item, paying particular attention to the positive-selection site table and the RNA-seq/expression table.

**4. Make the underlying data publicly accessible and provide a DOI.**  The data are currently shared via an anonymized, view-only OSF link. PLOS policy requires that the repository be publicly accessible at acceptance and cited by a persistent identifier. Please make the OSF project public, provide its DOI in the Data Availability Statement, and confirm that the alignments and tree files underlying Figures 1, 2, and 5 and Supplementary Figure 1 are all included, as committed in your responses.

**5. Calibrate two interpretive claims to the strength of the evidence.**  These do not require new analysis, only adjusted wording. (i) The proposed expansion of the alpharetrovirus host range into Afrotheria and rodents rests on BLASTx similarity (~45% amino-acid identity) from a single CHIRalphaEnv7 query; please ensure the Abstract's claim of expansion "into multiple mammalian clades" is phrased as a similarity-based inference rather than a demonstrated finding. (ii) The reproductive-function argument rests on two endometrium samples; your added limitation text is appropriate, and I would simply ask that the Abstract's "indicating an involvement in reproductive function" be softened (e.g., "suggesting") to match.

Please return a revised manuscript along with a point-by-point response describing how each item above has been addressed. Given the focused nature of these revisions, I anticipate making a decision without sending the manuscript out for another full round of review. I look forward to receiving the revised version.

Please submit your revised manuscript within by Jul 23 2026 11:59PM. If you will need more time than this to complete your revisions, please reply to this message or contact the journal office at plosgenetics@plos.org. When you're ready to submit your revision, log on to https://www.editorialmanager.com/pgenetics/ and select the 'Submissions Needing Revision' folder to locate your manuscript file.

Please include the following items when submitting your revised manuscript:

* A letter that responds to each point raised by the editor and reviewer(s). You should upload this letter as a separate file labeled 'Response to Reviewers'. This file does not need to include responses to formatting updates and technical items listed in the 'Journal Requirements' section below.

* A marked-up copy of your manuscript that highlights changes made to the original version. You should upload this as a separate file labeled 'Revised Manuscript with Track Changes'.

* An unmarked version of your revised paper without tracked changes. You should upload this as a separate file labeled 'Manuscript'.

If you would like to make changes to your financial disclosure, competing interests statement, or data availability statement, please make these updates within the submission form at the time of resubmission. Guidelines for resubmitting your figure files are available below the reviewer comments at the end of this letter.

As the corresponding author, your ORCID iD is verified in the submission system and will appear in the published article. PLOS supports the use of ORCID, and we encourage all coauthors to register for an ORCID iD and use it as well. Please encourage your coauthors to verify their ORCID iD within the submission system before final acceptance, as unverified ORCID iDs will not appear in the published article. Only the individual author can complete the verification step; PLOS staff cannot verify ORCID iDs on behalf of authors.

We look forward to receiving your revised manuscript.

Kind regards,

Harmit S. Malik

Academic Editor

PLOS Genetics

Justin Fay

Section Editor

PLOS Genetics

Aimée Dudley

Editor-in-Chief

PLOS Genetics

Anne Goriely

Editor-in-Chief

PLOS Genetics

**Additional Editor Comments (if provided):**

**Journal Requirements:**

If the reviewer comments include a recommendation to cite specific previously published works, please review and evaluate these publications to determine whether they are relevant and should be cited. There is no requirement to cite these works unless the editor has indicated otherwise.

1) Please ensure that the CRediT author contributions listed for every co-author are completed accurately and in full.

At this stage, the following Authors/Authors require contributions: Zhaobo Zhang. Please ensure that the full contributions of each author are acknowledged in the "Add/Edit/Remove Authors" section of our submission form.

The list of CRediT author contributions may be found here: https://journals.plos.org/plosgenetics/s/authorship#loc-author-contributions

2) Please provide an Author Summary. This should appear in your manuscript between the Abstract (if applicable) and the Introduction, and should be 150-200 words long. The aim should be to make your findings accessible to a wide audience that includes both scientists and non-scientists. Sample summaries can be found on our website under Submission Guidelines:

https://journals.plos.org/plosgenetics/s/submission-guidelines#loc-parts-of-a-submission

3) We do not publish any copyright or trademark symbols that usually accompany proprietary names, eg ©,  ®, or TM  (e.g. next to drug or reagent names). Therefore please remove all instances of trademark/copyright symbols throughout the text, including:

- TM on pages: 33, 34, and 35.

4) Please upload all main figures as separate Figure files in .tif or .eps format. For more information about how to convert and format your figure files please see our guidelines:

https://journals.plos.org/plosgenetics/s/figures

5) We are unable to open the following Supporting Information file: BatSupplementary.zip. Please kindly revise as necessary and re-upload.

6) Please amend your detailed Financial Disclosure statement. This is published with the article. It must therefore be completed in full sentences and contain the exact wording you wish to be published.

i) Please clarify all sources of financial support for your study. List the grants, grant numbers, and organizations that funded your study, including funding received from your institution. Please note that suppliers of material support, including research materials, should be recognized in the Acknowledgements section rather than in the Financial Disclosure

ii) State the initials, alongside each funding source, of each author to receive each grant. For example: "This work was supported by the National Institutes of Health (####### to AM; ###### to CJ) and the National Science Foundation (###### to AM)."

iii) State what role the funders took in the study. If the funders had no role in your study, please state: "The funders had no role in study design, data collection and analysis, decision to publish, or preparation of the manuscript."

iv) If any authors received a salary from any of your funders, please state which authors and which funders..

If you did not receive any funding for this study, please simply state: u201cThe authors received no specific funding for this work.u201d

7) We have amended your Competing Interest statement to comply with journal style. We kindly ask that you double check the statement and let us know if anything is incorrect.

**Reviewers' comments:**

[NOTE: If reviewer comments were submitted as an attachment file, they will be attached to this email and accessible via the submission site. Please log into your account, locate the manuscript record, and check for the action link "View Attachments". If this link does not appear, there are no attachment files.]

**Figure resubmission:**

**Reproducibility:**

To enhance the reproducibility of your results, we recommend that authors deposit laboratory protocols in protocols.io, where a protocol can be assigned its own identifier (DOI) such that it can be cited independently in the future. Additionally, PLOS ONE offers an option to publish peer-reviewed clinical study protocols. Read more information on sharing protocols at https://plos.org/protocols?utm_medium=editorial-email&utm_source=authorletters&utm_campaign=protocols

# Author comment: Author response to Decision Letter 1

**DOI:** 10.1371/journal.pgen.1012277.r003

21 Jul 2026

Attachment Submitted filename: Response to editor.pdf

# Editor report: Decision Letter 1

**DOI:** 10.1371/journal.pgen.1012277.r004

**Authors:** Justin Fay, Harmit Malik

2 Aug 2026

Dear Dr Katzourakis,

We are pleased to inform you that your manuscript entitled "An ancient alpharetrovirus lineage in bats: evolutionary insights and possible roles in reproduction" has been editorially accepted for publication in PLOS Genetics. Congratulations!

Before your submission can be formally accepted and sent to production you will need to complete our formatting changes, which you will receive in a follow up email. Please be aware that it may take several days for you to receive this email; during this time no action is required by you. Please note: the accept date on your published article will reflect the date of this provisional acceptance, but your manuscript will not be scheduled for publication until the required changes have been made.

Once your paper is formally accepted, an uncorrected proof of your manuscript will be published online ahead of the final version, unless you’ve already opted out via the online submission form. If, for any reason, you do not want an earlier version of your manuscript published online or are unsure if you have already indicated as such, please let the journal staff know immediately at plosgenetics@plos.org.

In the meantime, please log into Editorial Manager at https://www.editorialmanager.com/pgenetics/, click the "Update My Information" link at the top of the page, and update your user information to ensure an efficient production and billing process. Note that PLOS requires an ORCID iD for all corresponding authors. Therefore, please ensure that you have an ORCID iD and that it is validated in Editorial Manager. To do this, go to ‘Update my Information’ (in the upper left-hand corner of the main menu), and click on the Fetch/Validate link next to the ORCID field.  This will take you to the ORCID site and allow you to create a new iD or authenticate a pre-existing iD in Editorial Manager.

If you have a press-related query, or would like to know about making your underlying data available (as you will be aware, this is required for publication), please see the end of this email. If your institution or institutions have a press office, please notify them about your upcoming article at this point, to enable them to help maximise its impact. Inform journal staff as soon as possible if you are preparing a press release for your article and need a publication date.

Thank you again for supporting open-access publishing; we are looking forward to publishing your work in PLOS Genetics!

Yours sincerely,

Harmit S. Malik

Academic Editor

PLOS Genetics

Justin Fay

Section Editor

PLOS Genetics

Aimée Dudley

Editor-in-Chief

PLOS Genetics

Anne Goriely

Editor-in-Chief

PLOS Genetics

www.plosgenetics.org

BlueSky: @plos.bsky.social

----------------------------------------------------

Comments from the reviewers (if applicable):

----------------------------------------------------

**Data Deposition**

If you have submitted a Research Article or Front Matter that has associated data that are not suitable for deposition in a subject-specific public repository (such as GenBank or ArrayExpress), one way to make that data available is to deposit it in the Dryad Digital Repository. As you may recall, we ask all authors to agree to make data available; this is one way to achieve that. A full list of recommended repositories can be found on our website.

The following link will take you to the Dryad record for your article, so you won't have to re‐enter its bibliographic information, and can upload your files directly:

http://datadryad.org/submit?journalID=pgenetics&manu=PGENETICS-D-26-00624R1

More information about depositing data in Dryad is available at http://www.datadryad.org/depositing. If you experience any difficulties in submitting your data, please contact help@datadryad.org for support.

Additionally, please be aware that our data availability policy requires that all numerical data underlying display items are included with the submission, and you will need to provide this before we can formally accept your manuscript, if not already present.

----------------------------------------------------

**Press Queries**

If you or your institution will be preparing press materials for this manuscript, or if you need to know your paper's publication date for media purposes, please inform the journal staff as soon as possible so that your submission can be scheduled accordingly. Your manuscript will remain under a strict press embargo until the publication date and time. This means an early version of your manuscript will not be published ahead of your final version. PLOS Genetics may also choose to issue a press release for your article. If there's anything the journal should know or you'd like more information, please get in touch via plosgenetics@plos.org.

# Editor report: Acceptance letter

**DOI:** 10.1371/journal.pgen.1012277.r005

**Authors:** Justin Fay, Harmit Malik

PGENETICS-D-26-00624R1

An ancient alpharetrovirus lineage in bats: evolutionary insights and possible roles in reproduction

Dear Dr Katzourakis,

We are pleased to inform you that your manuscript entitled "An ancient alpharetrovirus lineage in bats: evolutionary insights and possible roles in reproduction" has been formally accepted for publication in PLOS Genetics! Your manuscript is now with our production department and you will be notified of the publication date in due course.

The corresponding author will soon be receiving a typeset proof for review, to ensure errors have not been introduced during production. Please review the PDF proof of your manuscript carefully, as this is the last chance to correct any errors. Please note that major changes, or those which affect the scientific understanding of the work, will likely cause delays to the publication date of your manuscript.

Soon after your final files are uploaded, unless you have opted out or your manuscript is a front-matter piece, the early version of your manuscript will be published online. The date of the early version will be your article's publication date. The final article will be published to the same URL, and all versions of the paper will be accessible to readers.

For Research Articles, you will receive an invoice from PLOS for your publication fee after your manuscript has reached the completed accept phase. If you receive an email requesting payment before acceptance or for any other service, this may be a phishing scheme. Learn how to identify phishing emails and protect your accounts at https://explore.plos.org/phishing.

Thank you again for supporting PLOS Genetics and open-access publishing. We are looking forward to publishing your work!

With kind regards,

Anitha Samidurai

PLOS Genetics

On behalf of:

The PLOS Genetics Team

Carlyle House, Carlyle Road, Cambridge CB4 3DN | United Kingdom

plosgenetics@plos.org | +44 (0) 1223-442823

plosgenetics.org | Twitter: @PLOSGenetics
